# Recent Trends in Metabolomics by NMR Spectroscopy

**DOI:** 10.1002/anie.202525689

**Published:** 2026-04-23

**Authors:** Giorgio Di Paco, Gaia Meoni, Veronica Ghini, Alessia Vignoli, Leonardo Tenori, Paola Turano, Claudio Luchinat

**Affiliations:** ^1^ Department of Chemistry, “Ugo Schiff” University of Florence Via della Lastruccia Sesto Fiorentino Florence Italy; ^2^ Magnetic Resonance Center (CERM) University of Florence Via Luigi Sacconi Sesto Fiorentino Florence Italy; ^3^ Consorzio Interuniversitario Risonanze Magnetiche di Metallo Proteine (CIRMMP) Via Luigi Sacconi Sesto Fiorentino Florence Italy

**Keywords:** literature analysis, metabolomics, NMR spectroscopy, research trends

## Abstract

This review is an update of our previous contribution published in *Angewandte Chemie* in January 2019 and provides a critical analysis of the overall scientific production and key findings from NMR‐based metabolomics between January 2018 and April 2025. We developed a strategy (described in the Methodological Approach section) for systematically analyzing the literature that enabled us to identify 5081 studies published during this observation period. Descriptive statistics were used to summarize this large dataset in terms of frequency distribution of certain experimental parameters (e.g., magnetic field, pulse sequences, NMR active nuclei, etc.) and to identify six broad main fields of application: human health, food and nutrition, veterinary, plants, environment and analytical methods. The major one remains human health; in absolute terms, its share is greater than the sum of the other five. Within each field, we focused on a few key topics and, for each of them, we provided a detailed and critical analysis of the three articles that have received the largest number of citations per year.

## Graphical abstract

AI tools were applied to analyze more than 5 000 publications indexed in Scopus (2018–2025), identifying key trends and research directions in NMR‐based metabolomics. The artificial intelligence‐assisted workflow classified papers into six main fields of application, human health, food and nutrition, veterinary science, plants, environment, and analytical methods, underlining the growing interdisciplinarity and maturity of NMR metabolomics across diverse scientific domains.

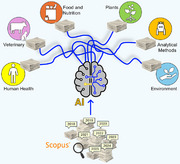

## 1 Introduction

Metabolomics has been around for about a quarter of a century, and its share of scientific literature has been steadily increasing, witnessing its success. Metabolomics is the science that—in principle—aims at studying the whole *metabolome*, as opposed to the study of single metabolites or groups thereof. In practice, due to limitations in our analytical abilities, the whole metabolome is hardly reachable. However, identification and quantification of a relatively large number of metabolites in a biological sample already provides precious information on that sample. Metabolomics is applied to biological samples from many different research fields: human health, plants and animals, food and nutrition, and environment being the most popular.

Metabolomics is mostly carried out by mass spectrometry (MS) and nuclear magnetic resonance (NMR) spectroscopy, with relative shares of about 85% and 15%, respectively—these shares being roughly constant over the years. There are many reasons for this imbalance, the main being the much larger diffusion of mass spectrometers in analytical laboratories, including in medical settings. In addition, MS is much more sensitive than NMR, thereby granting the possibility of detecting larger numbers of metabolites. Why then did metabolomics by NMR not yet fade away, and has instead established its own scientific area and distinct range of applications? At variance with MS, samples for NMR are easier to prepare, NMR analysis is straightforward and, most importantly, the results are intrinsically quantitative. This latter feature has prompted the development of quantitative methods, some of which have worked their way from academic research settings to industry‐developed analytical platforms; they are offered to customers as a commercial service and provide reliable results. It is believed that official recognition by regulatory agencies of NMR methods for specific in vitro diagnosis (IVD) is close to becoming a reality.

**FIGURE 1:**
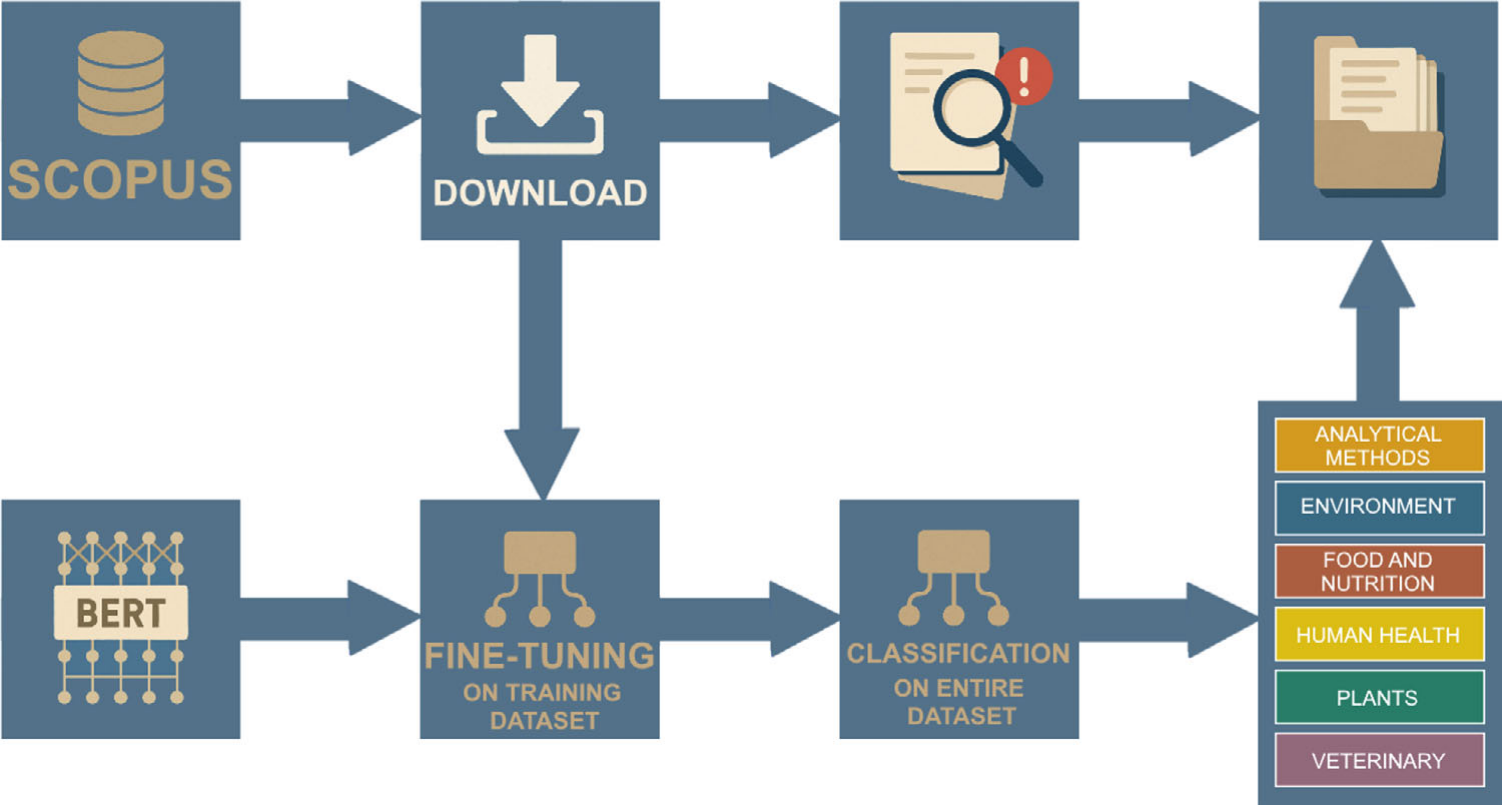
Workflow of the literature search strategy developed for this review. Schematic representation of the in‐house and AI‐based tools applied to retrieve and classify NMR‐based metabolomics papers according to year, research field, and methodological keywords. Details on each block are provided in the Methodological Approach section.

Metabolomic literature has been extensively reviewed in recent years, with different focuses, either technique‐centered (MS and/or NMR) [1, 2, 3, 4, 5] or application‐centered (e.g., human health) [6, 7, 8, 9, 10, 11, 12, 13, 14]. Some of us have reviewed NMR‐based metabolomics in *Angewandte Chemie* in 2019, with special emphasis on high‐throughput methods [15]. The present review could be considered an update of that work, but at the same time we decided to focus on trends, that is, trying to grasp how metabolomics by NMR has been evolving in the past seven years and what its perspectives are for the next few years. To do so, we have developed in‐house some literature search tools, tailored to metabolomics, also exploiting basic level artificial intelligence (AI) techniques, as summarized in Figure 1 and detailed in the methodological approach section. We have also faced the non‐trivial task of selecting keywords or, better, filtering out keywords contained in metabolomics papers that could have introduced biases in our research. At the end of the process, we have selected 5081 research articles. The following summary of our search results provides interesting hints to predict the evolution of NMR metabolomics in the next few years.

**FIGURE 2:**
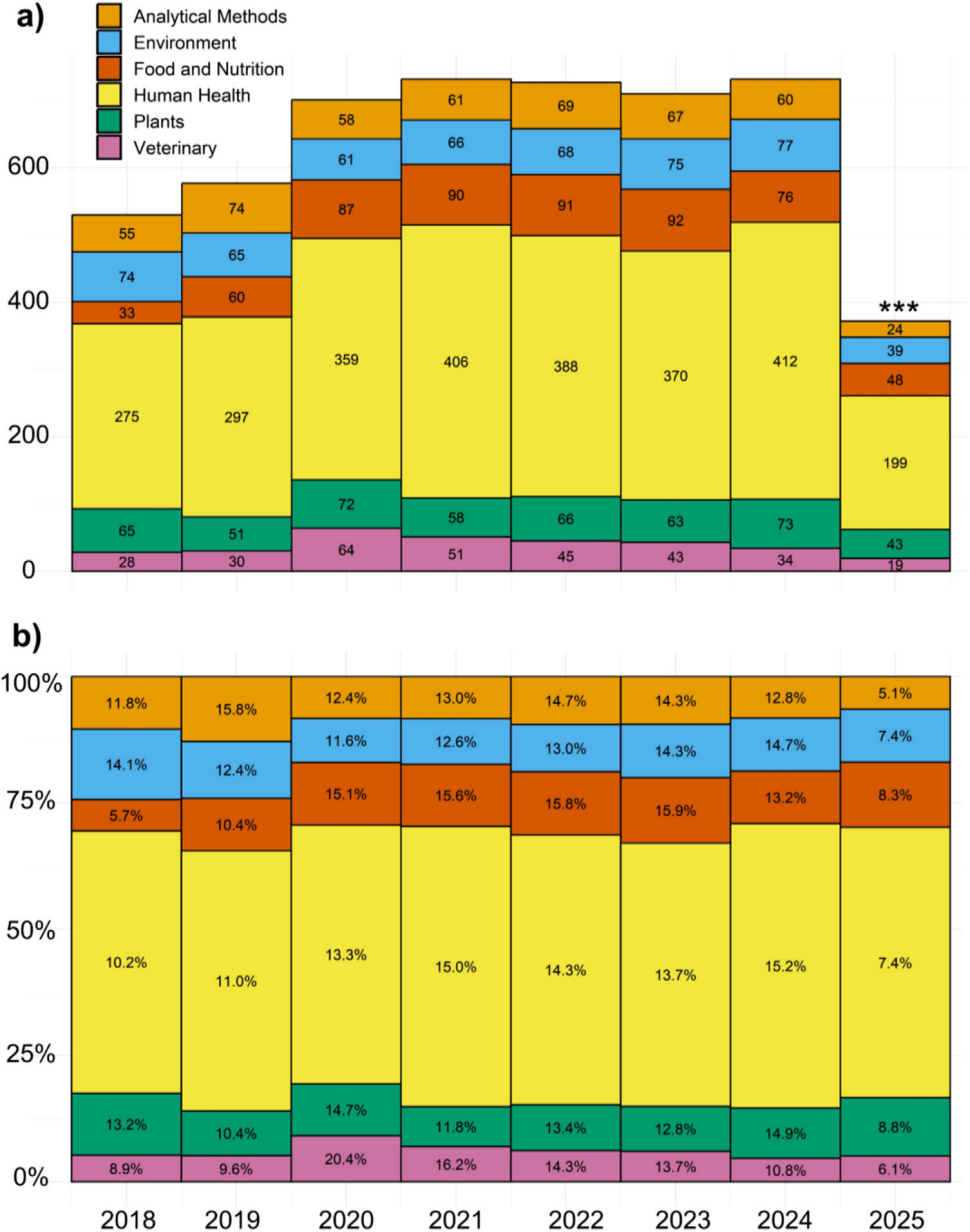
Number of NMR‐based metabolomics publications in the period 2018–2025. a) Number of papers per year, divided according to the main fields (numbers represent absolute values); ***: for 2025 the numbers are limited to the period Jan–Apr. b) Percentage of papers in each field per year (numbers represent percentage values).

We immediately noted that the number of published papers has leveled off starting from 2020 (Figure 2a). The number of papers that combine MS with NMR has been increasing in the last years. Our systematic survey indicates while MS was employed in approximately 30% of the total selected articles, its prevalence is strictly context‐dependent. Veterinary exhibit the lowest integration of MS with NMR (14%), followed by human health (25%). A compelling contrast is observed in plant science, where the reliance on the combined approach is much higher (53%). This trend reflects the inherent analytical challenges posed by different biological matrices. In disciplines characterized by vast chemical complexity, such as plant metabolomics, MS is often essential for unambiguous metabolite identification. Furthermore, in scenarios necessitating extensive sample manipulation and extraction protocols, the traditional hallmark of NMR—its minimal sample pre‐treatment—is effectively nullified. Consequently, the superior sensitivity of MS overshadows the procedural simplicity of NMR for comprehensive molecular profiling in complex systems. It will be interesting to monitor these trends in the coming years to better understand the evolution of the research area.

It is also interesting to see how the main metabolomic fields (human health, food and nutrition, veterinary, plants, and environment) tend to evolve (Figure 2a). The increase in the total number of publications observed between 2018 and 2020 reflects an increase in all abovementioned five fields except environment. Having grouped methodological papers into a sixth field, we note that methodological studies have instead not significantly increased since 2019. This could be an indication that NMR‐based metabolomics is a mature science and that methodological developments are less required than in the past.

**FIGURE 3:**
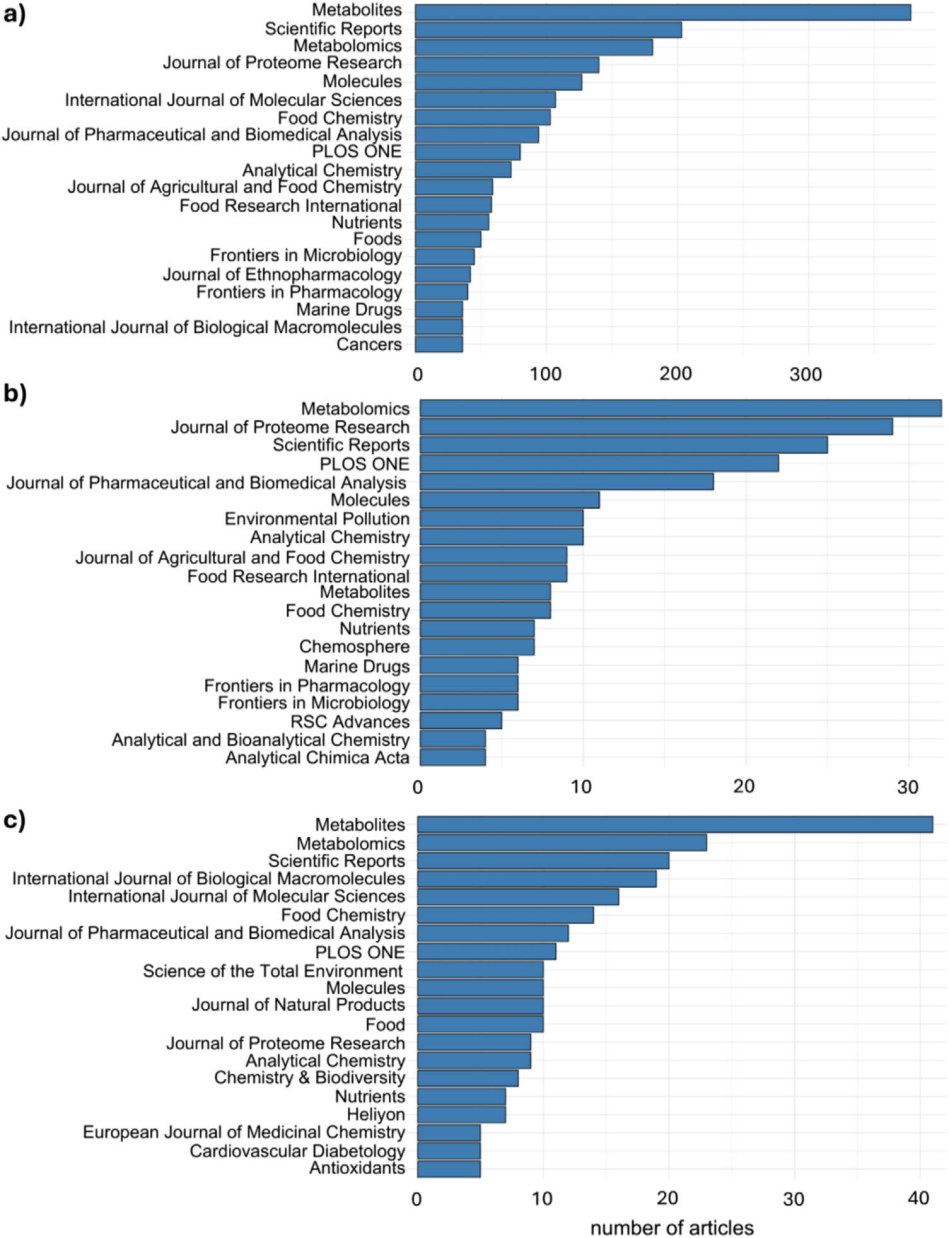
Top 20 journals in terms of the number of NMR‐based metabolomics articles published. (a) in the period 2018–2025; (b) in 2018; (c) in 2024.

Also, while after the growth between 2018 and 2020 the number of human health studies has stabilized, we can see that food and nutrition, and plants have increased their share (Figure 2b). This is also reflected in the partitioning of metabolomic papers in the most representative journals for the field (Figure 3). From Figure 3c it can be immediately seen that new journals have entered the list, among which *Metabolites* has notably jumped from the eleventh to the first place. We also note a decrease of *Journal of Proteome Research*, a more generic “omic” journal, while journals related to food, nutrition, plants, and animals have collectively increased their share by about 30%.

**FIGURE 4:**
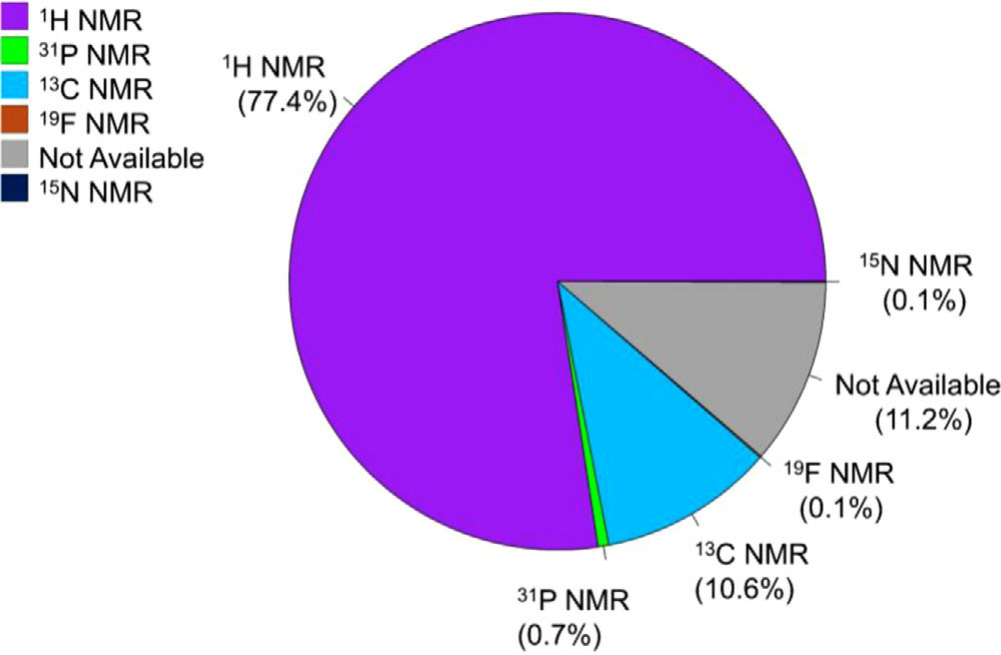
Pie chart illustrating the numerical proportion of the various NMR nuclei used in the selected metabolomics studies. All 2D experiments involving heteronuclei were attributed to the heteronucleus category regardless of whether direct or inverse detection was employed.

**FIGURE 5:**
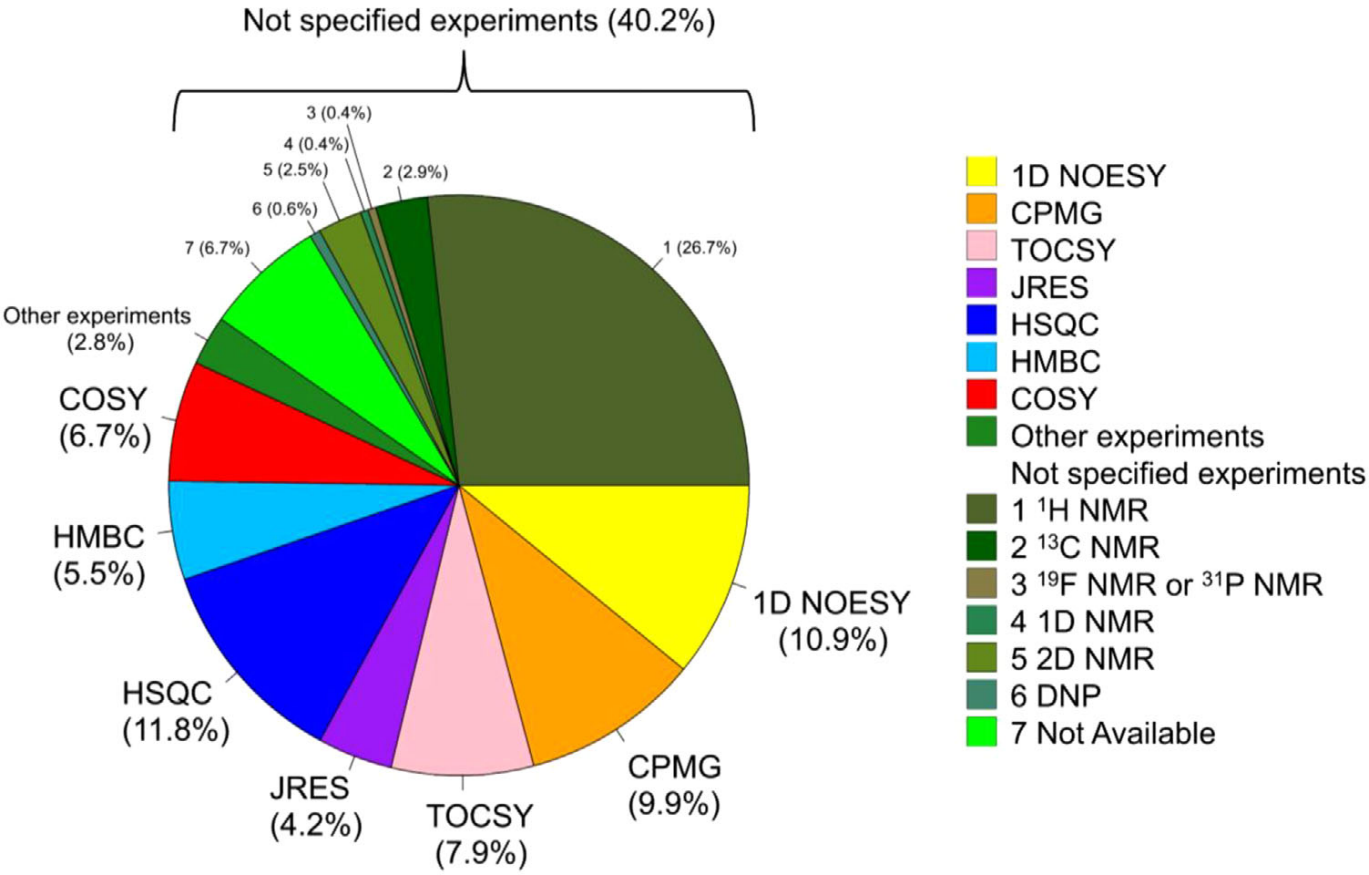
Pie chart of the NMR experiment types used in the selected metabolomics studies. The percentages reported were obtained by counting all NMR experiment types mentioned in each publication.

In terms of methodology, Figure 4 shows that, as expected, metabolomic studies are largely performed by ^1^H NMR, with ^13^C NMR being the only other relevant nucleus, used often to study metabolic fluxes. ^31^P and ^19^F nuclei are much less used. Figure 5 shows the most commonly used experiments, all of them obviously dealing with pure ^1^H NMR spectroscopy or with ^1^H‐^13^C bidimensional experiments. In standard natural abundance metabolomics, due to its low sensitivity, ^1^ ^3^C NMR is almost exclusively used in ^1^H detected experiments to take advantage of its wide chemical shift range. In the case of ^15^N, detection is further complicated by exchange phenomena affecting the signals of NH, NH_2_, and NH_3_ as a function of pH and temperature; consequently, its contribution is essentially negligible. ^31^P NMR can further expand the NMR accessible metabolome but one must cope with several challenges associated with the heavy pH and temperature dependence of the chemical shifts, and poor line shapes affected by ionization state and metal ions concentrations. Finally, ^19^F can be used to trace fluorinated compounds with high sensitivity and no background interference.

**FIGURE 6:**
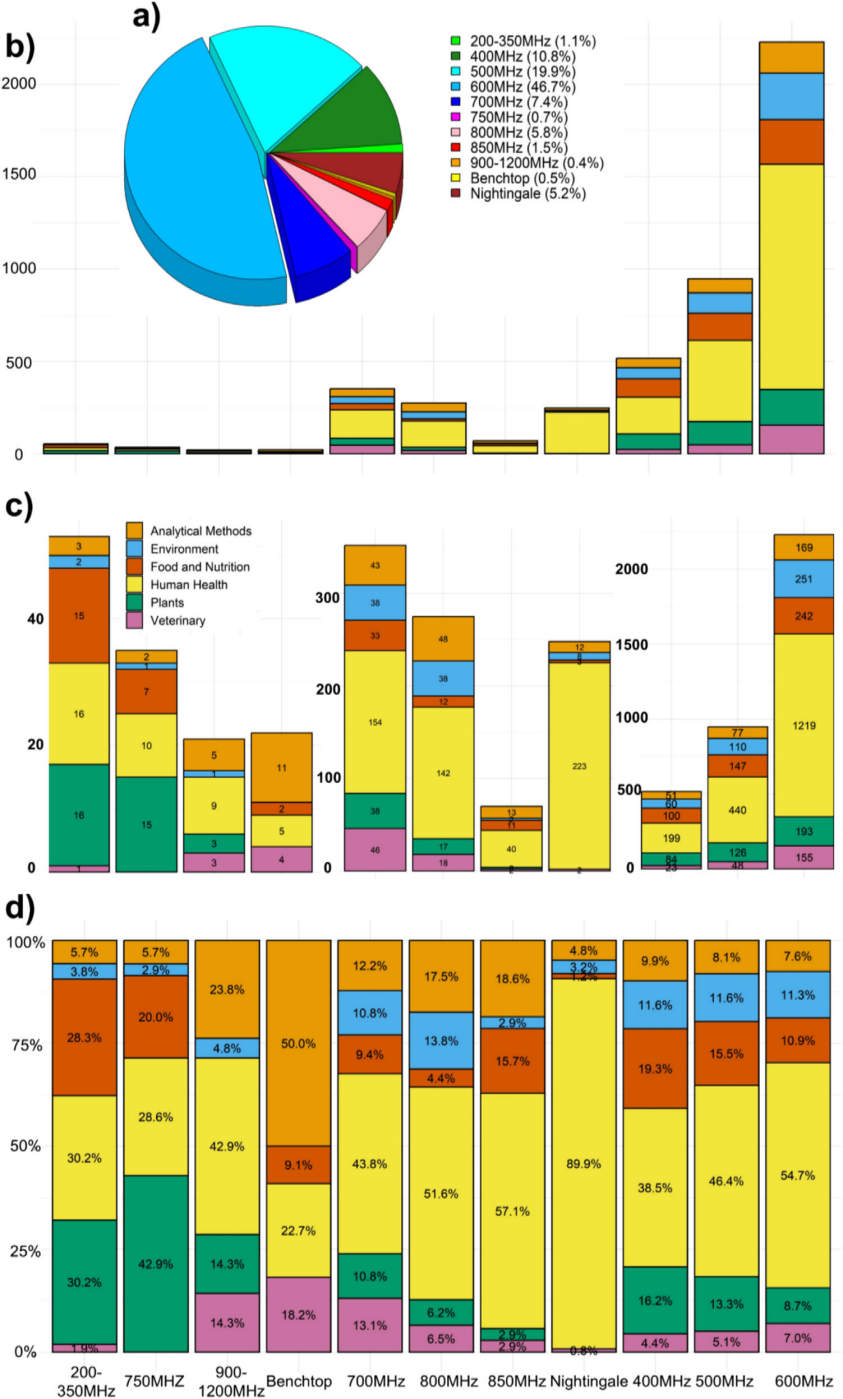
Distribution of NMR platforms used in the selected studies, including benchtop spectrometers (grouped into a single category) and the Nightingale service (for which the acquisition magnetic field is not reported in the corresponding studies). (a) Pie chart representation of the overall distribution of platforms, (b) absolute numbers of papers using each platform. (c) Platforms grouped into tertiles according to the total number of articles, to enhance visualization of both highly and poorly represented frequency ranges, with bars reporting the absolute number of articles. (d) Percentages of papers using the various platforms, divided according to the main research fields.

As of today, 600 MHz instruments continue to be the most used instruments in metabolomics (Figure 6a). This prevalence is mainly due to the leading instrument manufacturer recommending, for human health studies, 600 MHz instruments as the best compromise between sensitivity and resolution on the one hand and cost on the other. A large share is also occupied by 500 and 400 MHz instruments; the latter being recommended for food studies (Figure 6b,c).

**FIGURE 7:**
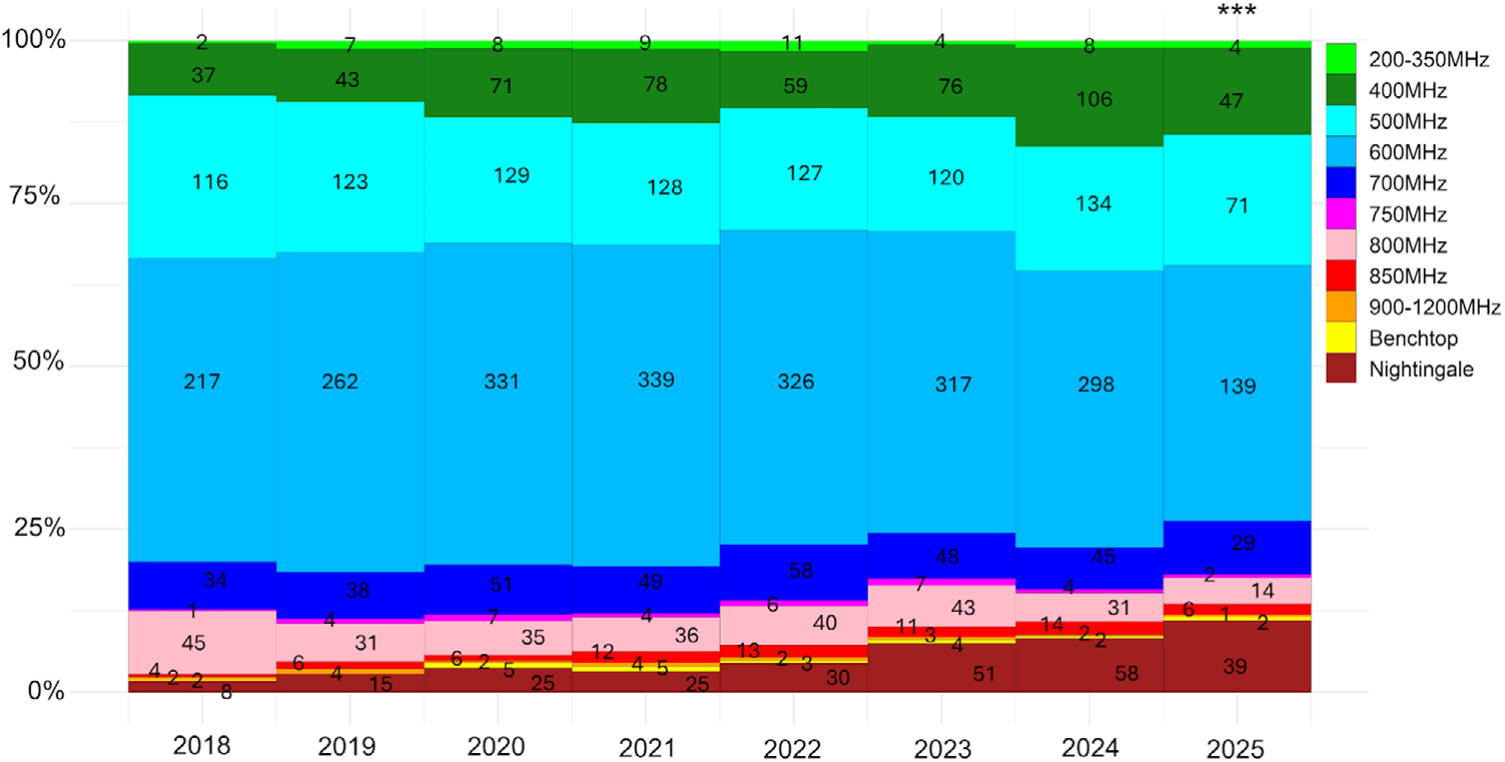
Percentages of papers using the various NMR‐based platforms over the years 2018–2025. For each year, the number of papers in each strip is reported. ***: for 2025 the numbers are limited to the period Jan–Apr.

However, if we look at the overall distribution of magnetic fields over the last six years (Figure 7), there is a tendency to go to lower fields, even benchtop instruments (although not much represented yet), which may become important for specific analyses once they are more standardized. A separate comment is due to the “Nightingale” category. Nightingale is a company that provides NMR metabolomics services, mainly for human health, and the results of the Nightingale analyses often do not specify the magnetic field at which they were obtained. In any case, the growth of the studies using this service (Figure 7) witnesses that metabolomics is becoming a routine analysis that researchers are confident to outsource to a private company. Interestingly, Nightingale is almost exclusively focused on human health research.

Hereafter, we present the most cited examples identified for the six metabolomics fields of Figure 2 (i.e., analytical methods, human health, food and nutrition, veterinary, plants, and environment). For each field, we have identified selected sub‐topics on subjects that to us seem particularly relevant; within each sub‐topic, we discuss in detail three articles among those with the highest average number of citations per year, resulting in a core selection of 42 articles analyzed in depth. This approach was chosen because different topics/sub‐topics have inherently different citation densities and audience sizes. Specifically, at the moment of our selection, the selected articles fall within a range of 9 (Plants) to 49 (Analytical methods) average citations per year. By prioritizing the annual citation rate, the selection naturally emphasizes established high‐impact contributions. Consequently, 2025 publications were excluded as they have not yet accrued a citation record. Nevertheless, this criterion allowed for a balanced sampling across the seven‐year period analyzed (2018–2024): notably, 12 out of the 42 selected works belong to the most recent triennium (2022–2024), while the overall distribution shows a citation peak in the 2020–2021 biennium.

## 2 Analytical Methods

The metabolomics workflow comprises various steps that can be summarized in three macro‐phases: pre‐analytics, NMR analysis, statistics & model building, which we have collectively grouped under the category of *Analytical Methods*.

The pre‐analytical phase consists of two main activities: i) selecting the appropriate number of samples for testing and validation; ii) collecting the most appropriate sample type and using suitable standard operating procedures to preserve the original metabolome composition as much as possible. The former activity is particularly critical when analyzing human (and animal) samples, as it enables significant signatures to be extracted for a given pathophysiological state or to track the effects of medical intervention, provided it is associated with adequate demographic and clinical information.

The NMR analysis involves preparing the sample for the NMR experiment, acquiring the spectra, and processing them to compile the data to be used in the subsequent phase of interpretation of the results. This phase is crucial for ensuring the technical reliability of the results.

In the third and final stage, a variety of statistical methods are employed to extract significant signatures and biomarkers, and to create mathematical models that describe the relationships between variables.

We and others have extensively reviewed the problems and solutions available for the various steps and for a large variety of samples [3, 15, 16, 17, 18, 19, 20]. Hereafter, we have reviewed some among the top articles to identify the cutting‐edge research in terms of methods.

### 2.1 Cohort and Sample Selection

The work by Buergel et al. [21] is a striking example of the importance of cohort size to obtain significant disease‐specific NMR‐derived metabolomic profiles of potential clinical utility (either as a replacement or as an additional source of discriminatory information to refine comprehensive risk assessments). Predictors and disease endpoints were extracted by serum NMR profiling at the time of cohort recruitment for 117 981 individuals in the UK Biobank population cohort, from 22 recruitment centers. The recruitment period was 2006–2010 and follow‐up ongoing. The NMR analysis provided 168 molecular components including lipoproteins, used to model the integrative metabolomic state for 24 common diseases simultaneously (i.e., major adverse cardiac event, coronary heart disease, cerebral stroke, dementia, heart failure, atrial fibrillation, type 2 diabetes, liver disease, renal disease, peripheral artery disease, venous thrombosis, abdominal aortic aneurysm, chronic obstructive pulmonary disease, asthma, Parkinson's disease, cataract, glaucoma, fracture, lung cancer, skin cancer, colon cancer, rectal cancer, prostate cancer, and breast cancer). Thanks to the availability of rich longitudinal health records in the cohorts analyzed it was possible to systematically investigate whether metabolic profiles could be used to predict the onset of diseases. All individuals from a single center were retained for testing models that were trained on individuals pooled from the 21 remaining recruitment centers; the validation set was based on randomly selecting 10% of the aggregated partitions. Finally, external validation was performed in four independent cohorts (Whitehall II, 6117 individuals; Rotterdam study, 2949 individuals; Leiden longevity, 1655 individuals; PROSPER cohort, 960 individuals). The predictive information of the NMR metabolomics assay was investigated against common clinical variables and resulted to be predictive for all the investigated diseases except for breast cancer. Often, the metabolomic discriminatory information was found to be shared with established clinical predictors, but for several endpoints, including type II diabetes, all‐cause dementia and heart failure, it contained complementary information that added predictive value [21]. As in the example above, serum and blood plasma are the most used biofluids in human health studies. They have several advantages: a high degree of homeostatic stability due to multiple, redundant feedback systems that maintain a dynamic equilibrium in the blood with respect to other biofluids (such as urine and saliva), which are more prone to daily and circadian variability; the collection procedure is minimally invasive; they are the most common bio‐banked biospecimens. The choice of serum or plasma, and of plasma collection tubes, has been the subject of several studies, including those by Sotelo‐Orozco et al. [22] and Vignoli et al. [23] These studies have highlighted the main differences in terms of the stability of various molecules, and the “contamination” of spectral profiles induced by anticoagulants and additives in the tube. The former study focused primarily on differences in approximately 50 small‐molecule metabolites, using samples from eight healthy female volunteers and six different tubes, including plastic tubes with no additives for serum, and tubes containing acid citrate dextrose, sodium citrate, EDTA, sodium fluoride and sodium heparin for plasma. The mean differences and percent coefficient of variation of metabolite concentrations between serum (taken as the control) and each plasma tube type were calculated to compare differences in specific metabolite concentrations between tubes [22].

In the latter study, the authors extended their analysis to include lipoproteins, in addition to the quantification of 34 metabolites, using three different blood collection tubes: citrate plasma tubes, EDTA plasma tubes and serum tubes. They also proposed a regression‐based statistical solution that can efficiently mitigate the impact of different collection tubes on both metabolomic and lipoprotein profiles. This approach is valuable for rescaling metabolomics and lipoprotein levels for use in epidemiological studies based on samples from multicentric cohorts that have used different collections methods [23].

### 2.2 Novel Approaches for Spectral Acquisition and Processing

To date, the intrinsic low sensitivity of the technique has been one of the most critical aspects of NMR‐based metabolomics. Simply switching from a standard 600 MHz instrument to a top 1.2 GHz instrument does not significantly increase the number of metabolites above the detectability threshold [16]. A significant variation in threshold can be achieved with dynamic nuclear polarization (DNP). For example, Dey et al. [24] advanced the sensitivity by exploiting hyperpolarization: they reported an improved pipeline for untargeted metabolomic approach using dissolution DNP (d‐DNP) on ^13^C natural‐abundance samples. Because ^13^C has much wider chemical shift dispersion than ^1^H, it is attractive for metabolomics, but its low sensitivity has prevented its routine use. Dey et al. overcame this by hyperpolarizing ^13^C in a cryogenic solid state, dissolving the sample rapidly, and then acquiring ^13^C NMR in one or a few scans. They showed that their workflow distinguishes two groups of tomato extracts (red vs. green) in a principal component analysis (PCA) of bucketed ^13^C spectra, consistently with prior ^1^H‐based metabolomics. They also optimized parameters of the semi‐automated d‐DNP injection and relaxation protocols to reduce polarization loss and improve reproducibility. The strength of the approach lies in the dramatically enhanced sensitivity that makes ^13^C NMR feasible even in real biological mixtures. The main limitation is the high technical complexity and the requirements of specialized apparatus and skills. Further, reproducibility across experiments is still challenging.

Another critical aspect in NMR‐based metabolomics is resolution, which becomes increasingly important as the sample becomes richer in metabolites. In this sense urine represents one of the most challenging samples. In this regard, Li et al. [25] tackled one of the most persistent bottlenecks: accurate and consistent peak picking and deconvolution in crowded spectra [26]. Their method, DEEP Picker, is a convolutional deep neural network with eight hidden layers, trained on large synthetic spectral datasets of known composition, designed to deconvolute overlapping cross‐peaks even in challenging spectral regions, both in 1D and 2D spectra. The authors demonstrated its performance in urine, showing that DEEP Picker can reliably extract peaks even in regions with strong overlap. Because 2D spectra offer greater chemical‐shift dispersion and help resolve overlapped ^1^H resonances, DEEP Picker steps toward more routine 2D NMR metabolomics. A strength is its fully trained neural network architecture, allowing subpixel estimation of peak centers, widths, and Lorentzian/Gaussian proportions. Limitations include that training was limited to synthetic data and relies on a priori assumptions of chemical shifts, peak shapes, and noise models that may deviate in real biological spectra. Further, tests on serum/plasma 1D NOESY spectra (notoriously complicated by the presence of broad peaks due to macromolecules) were lacking. Nonetheless, DEEP Picker marks a significant step toward automating what has long been a labor‐intensive human task.

Instead, Khakimov et al. [27] focused on the challenge of converting raw ^1^H NMR urine spectra in interpretable data. Their method, signature mapping (SigMa), is a pipeline that transforms raw spectra into metabolite‐level “signature maps” by combining statistical filtering and matching to reference libraries. It derives from, and improves on, the well‐known icoshift [28] alignment algorithm. The goal is to bypass the usual binning procedure [29] and instead decompose the spectra into segments of three different kinds: quantified metabolites for unambiguous regions; integrated areas for clear but unassigned signals; and binned segments for complex and unresolved regions. In practice, they applied SigMa to large‐scale human urine spectra and showed that, thanks to its curated library, it was able to assign a large portion of the urine NMR spectra without heavy manual intervention, and without discarding information embedded in unassigned regions. In practice, it combines the strengths of “fingerprinting” and “profiling” approaches [16], allowing both biological interpretation and a full input for multivariate models. The authors benchmarked SigMa's performance in human urine in comparison to both standard binning and full point‐by‐point spectra and found generally stronger statistical models when using SigMa‐derived features (about 40 metabolites, 50 unknown peaks, and 60 bins). A limitation of the approach is that it depends on ad hoc curated libraries and cannot always resolve metabolites in overlapping regions, where misassignment remains possible. Moreover, being specifically designed for the analysis of urine 1D NOESY spectra, it cannot be applied to other kinds of spectra and biofluids.

Viewed as a whole, these three studies represent complementary aspects of the evolving NMR metabolomics methods. DEEP Picker addresses the foundational problem of spectral deconvolution [26]: if you can't reliably pick peaks, the following assignment and quantification are useless. SigMa sits at the interface between raw spectra and metabolite‐level interpretation, and hyperpolarized ^13^C NMR pushes the frontier of detection by enhancing resolution (thanks to ^13^C detection) and sensitivity (thanks to d‐DNP). Together, they depict a pipeline in evolution, and in practical terms, these tools could be integrated: deep learning enables reliable deconvolution (DEEP Picker) and makes spectral complexity tractable; feature‐mapping approaches (SigMa) turn signals into usable metabolite‐level inputs; and hyperpolarization (^13^C d‐DNP) addresses the sensitivity limits of NMR. The challenge for the field now is to integrate new advances into robust pipelines and validate them across biological samples and conditions.

### 2.3 Databases

Metabolite assignment provides the components that form the basis of any metabolic model. That is why availability of curated databases is particularly valuable. These databases serve as platforms for spectral matching, metabolite identification, and interpretation of the biochemical context that links identified small molecules to physiology and diseases. The three resources discussed here: HMDB (Human Metabolome Database), DrugBank, and MiMeDB (the Human Microbial Metabolome Database), occupy complementary niches in this ecosystem, and together illustrate how spectral, pharmacologic, and microbial information can be converted into interpretable metabolomic data.

HMDB [30] (https://www.hmdb.ca/about) is probably the most mature and widely used metabolomics database. It includes not only metabolite chemical structures and pathways but also quantitative concentration ranges in different biofluids, associated diseases, and, crucially for NMR metabolomics, spectral data (1D and selected 2D references) and sample‐condition metadata. The strength of HMDB lies in its breadth: it houses tens of thousands of metabolite entries, extensively cross‐linked to external resources (KEGG, ChEBI, UniProt, etc.), and supports NMR (and MS) spectral search and matching capabilities. Because every “MetaboCard” entry includes rich biochemical and clinical metadata, HMDB acts as a sort of generalist reference hub.

DrugBank [31] (https://go.drugbank.com/) complements HMDB by bringing a pharmacological dimension that HMDB does not emphasize. DrugBank curates approved drugs, potential drugs, metabolites of drugs, and extensive pharmacokinetic information. For NMR metabolomics this is very useful whenever the spectra contain signals from pharmaceuticals, drug metabolites, or chemicals from environmental exposure: a search in DrugBank can distinguish endogenous metabolites from exogenous compounds and reveal possible biomarkers of drug metabolism. DrugBank's detailed annotations help researchers interpret how a detected small molecule might reflect medical treatment or environmental pollution. Because DrugBank is drug‐centric, the coverage of endogenous small molecules is limited only to those molecules that have drug‐related applications (e.g., alanine is present because it is *“an amino acid commonly found as a component of total parenteral nutrition”*).

MiMeDB [32] (Human Microbial Metabolome Database), instead, is rich in biological integration: it links microbes to the metabolites they produce, and further connects those to the human exposome and health. It includes over 24 000 metabolites linked with annotated microbial genomes, enzymatic reactions, and both MS and NMR spectral data for many of the microbial metabolites. Because many disease‐relevant metabolites derive from microbial metabolism (e.g., short‐chain fatty acids, secondary bile acids), MiMeDB fills a niche that general human‐centric metabolite databases often neglect. Its design allows users to ask: which microbe plausibly produced this metabolite, via which enzymatic route, and what is its known or predicted impact on host biology? This makes it especially powerful in microbiome–metabolome studies.

In short, HMDB, DrugBank, and MiMeDB represent complementary pillars for NMR metabolomics (Table 1). The next step, which is now within reach, is to integrate and interface tightly these databases with analytical software, creating an interoperable data flow that will shift NMR metabolomics from tedious manual matching to semiautomated, context‐aware annotation pipelines.

**TABLE 1:** Comparative information on GABA (γ‐Aminobutyric acid) across HMDB, DrugBank, and MiMeDB. This table illustrates how three databases provide complementary views of GABA, a key neurotransmitter, metabolite, and microbially‐ produced compound.

Database	Entry ID	Available Information	Key Strengths & Notes
HMDB	HMDB0000112	**Chemical & Structural Data**: IUPAC name, formula (C_4_H_9_NO_2_), SMILES, InChI, molecular weight, taxonomy (amino acids, neurotransmitters). **Spectral Data**: Experimental and predicted NMR spectra (^1^H, ^1^ ^3^C), MS reference spectra. **Biofluid & Tissue Distribution**: Quantified levels in plasma, cerebrospinal fluid, brain, urine, saliva. **Pathways**: Glutamate decarboxylation, GABA shunt. **Enzymes**: GAD1/GAD2 (glutamate decarboxylases), ABAT (GABA transaminase). **Associated Disorders**: Epilepsy, anxiety, hepatic encephalopathy.	HMDB provides rich biochemical and analytical data, linking structural identity, NMR features, and physiological relevance. Ideal for metabolite identification and validation in NMR studies.
DrugBank	DB01577	**Pharmacological Data**: Approved small molecule, occasionally used as dietary supplement. **Mechanism of Action**: Inhibitory neurotransmitter; acts on GABA‐A and GABA‐B receptors. **Pharmacokinetics**: Poor blood brain barrier penetration, known oral bioavailability. **Targets**: GABA‐A receptor complex, GABA‐B receptor. **Interactions**: With benzodiazepines, barbiturates, ethanol. Metabolism: From glutamate, degraded via GABA transaminase.	DrugBank adds pharmacological, receptor, and interaction data that connect biochemical roles with therapeutic context. Essential for linking metabolite behavior with neuroactive drug action.
MiMeDB	MMDBc0000632	**Chemical Data**: Formula, SMILES, synonyms. **Microbial Context**: Produced by gut microbes (Lactobacillus brevis, Bifidobacterium dentium, Escherichia coli). **Metabolic Routes**: Glutamate → GABA via microbial glutamate decarboxylase. **Health Context**: Implicated in gut‐brain axis and probiotic mechanisms. **Cross‐links**: HMDB, KEGG, PubChem.	MiMeDB adds microbial origin and biotransformation routes, giving context to host‐microbe metabolite exchange.

## 3 Human Health

**FIGURE 8:**
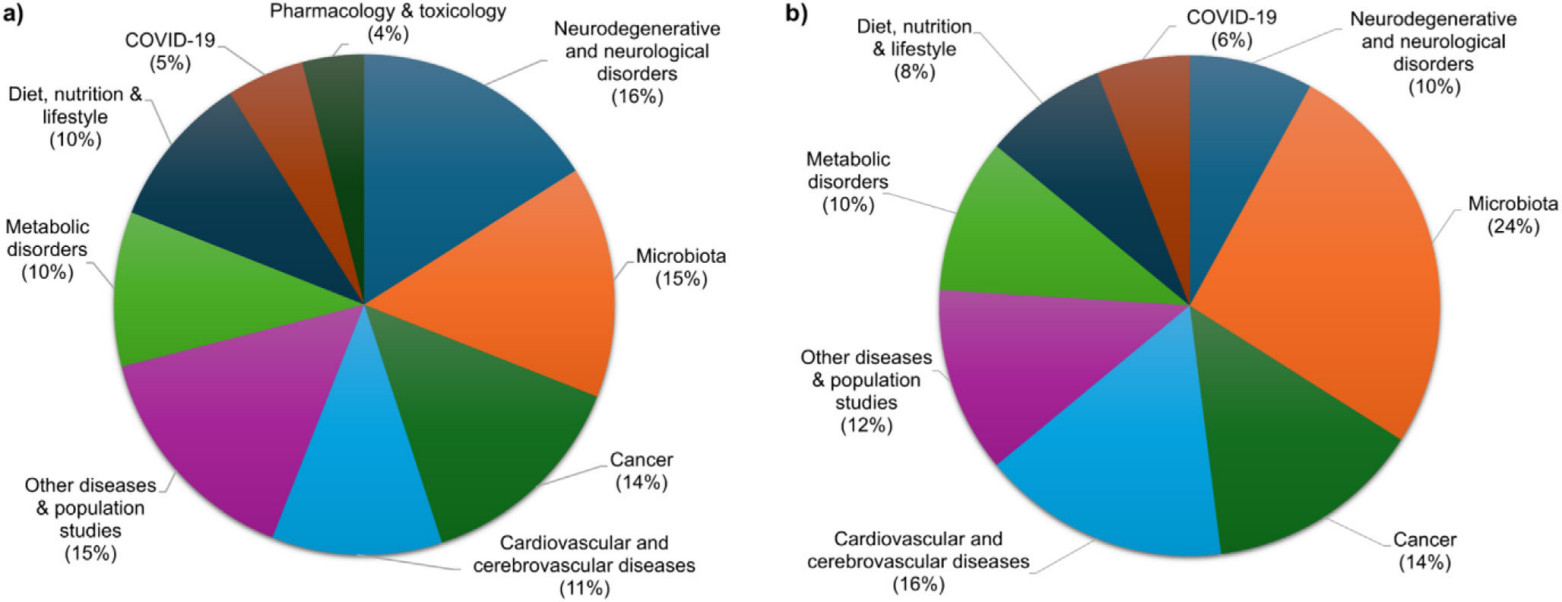
Distribution of research areas in the 2706 articles included in the “human health” category (Figure 2) represented as pie charts: (a) all 2706 analyzed articles (for this specific case, the classification of the data was performed using the Gemini artificial intelligence system, version 2.5 pro); (b) the top 50 articles with the highest number of citations per year.

As outlined in the Introduction, research activities relating to human health were quantitatively dominant during the period from 2018 to 2025 (Figure 2). Several key issues can be identified within this broad subject area, as summarized in Figure 8. Excluding the very generic category of “other diseases and population studies”, the top topics are: neurodegenerative and neurological disorders (particularly in relation to ageing), and analysis of the microbiota in human health, cancer, and cardiovascular and cerebrovascular diseases (Figure 8a). These topics remain at the forefront, even when analyzing the most frequently cited studies (Figure 8b), with microbiota jumping from 15% to 24%. Since 2020, metabolome analysis of samples from patients with severe acute respiratory syndrome Coronavirus 2 (SARS‑CoV‑2) infection has emerged as a relevant subject of interest. As in many other research areas, Coronavirus disease 2019 (COVID‐19) has attracted attention in metabolomics during the pandemic, with the international scientific community making major efforts to characterize this disease.

Based on the above considerations and the fact that under “cancer” falls a largely diverse group of diseases, we have decided to focus on the most significant examples of the four different fields detailed below.

### 3.1 Microbiota

In the last decades there has been increasing general interest in the role of the gut microbiota in affecting the host phenotypes and its association with various aspects of human health and disease [33]. In this framework, NMR‐based metabolomics has been established as a key platform for profiling gut microbiota‐related chemical signatures and monitoring their dynamic changes in both human and animal models. Metagenomic sequencing serves as a valuable complement, enabling detailed profiling of bacterial populations that can be correlated with the observed metabolomes. Here, we present three relevant examples of the metabolomics/metagenomics integration applied to different diseases. Further examples will be provided in the next paragraphs, where the focus is however mainly on the disease‐associated metabolic signatures.

The influence of the gut microbiota on the host metabotype is particularly pronounced in faecal samples, making them the most widely used sample types in this area of research in the form of aqueous extracts. The faecal metabolome provides a direct report of the metabolic activity of the gut microbiota and its interactions with the host, and it can be used for monitoring a wide range of physiological and pathological processes, such as response to diet or therapeutics and environmental or disease‐related perturbations [34, 35, 36]. A relevant example is provided by the study of Botticelli et al. where ^1^H NMR spectroscopy was applied to faecal samples from a small cohort of non‐small cell lung cancer patients undergoing second‐line immunotherapy (with nivolumab) [37]. This analysis allowed the quantification of 49 metabolites, including short‐chain fatty acids, amino acids, amines, and sugars. Metabolites such as propionate, butyrate, lysine, and nicotinic acid were associated with long‐term beneficial responses to immunotherapy, while others, including 2‐pentanone and tridecane, correlated with early disease progression. The NMR‐derived information on non‐volatile metabolites was integrated in a multivariate chemometric model with data from GC–MS on volatile metabolites, highlighting the possible contribution of the microbiome to the metabolomic signature associated with the responses to immunotherapy [37]. The interpretation in terms of microbiota provides a key to understanding previous observations on the value of blood metabolome as a “collective biomarker” for the responsiveness to immunotherapy in non‐small cell lung cancer [38].

A strong signature of gut microbiota‐derived metabolites has been also monitored in biofluids, making blood derivatives and urine also very commonly used sample types. Population‐based studies often aim at correlating gut‐microbiota characteristics with human diseases, such as cardiovascular or metabolic diseases [39]. For example, Aron‐Wisnewsky et al. explored the association of non‐alcoholic fatty liver disease with serum ferritin and gut microbiome [40]. In that work, ^1^H NMR spectroscopy was applied to plasma and urine samples from more than 350 subjects to characterize systemic metabolic alterations associated with both hepatic fat accumulation and microbial composition. Using a 600 MHz spectrometer, the authors identified several metabolites associated with serum ferritin and directly linked to the gut microbiome, including sarcosine, glutamate, glutamine, alanine, glycerol, histidine, tyrosine, glycine, and ketone bodies such as 3‐hydroxybutyrate and acetoacetate. The study revealed that the host iron status modulates the gut microbiota composition, which in turn alters the host's circulating metabolite profile, linking microbial metabolic function with hepatic lipid accumulation [40].

Vojinovic et al., using ^1^H NMR profiling of plasma samples (Nightingale platform) from two large population‐based cohorts (for a total of 2309 individuals), demonstrated a significant association of several microbial families and genera not only with circulating metabolites, such as glycolysis‐related molecules and ketone bodies, but also with lipoproteins. In particular, the gut microbiota composition showed a strong association with VLDL and HDL particles of various sizes, but a weak association with LDL and IDL particles, suggesting that the gut microbiota affects distinct classes of lipoproteins [41].

### 3.2 COVID‐19

The three most cited papers from our selection address different aspects in which NMR has been used to study COVID‐19 since the beginning of the pandemic: (i) characterization of the metabolomic signature of the disease, (ii) evaluation of treatment responses, and (iii) assessment of vaccine response.

The work of Bruzzone et al. [42] is representative of several studies in which ^1^H NMR fingerprinting of serum or plasma samples has been used to unambiguously describe the metabolomic signature of the disease [42, 43, 44, 45, 46, 47, 48, 49, 50, 51, 52, 53, 54]. The extensive reshaping of the serum metabolome and lipoproteome upon infection was described using 398 acute‐phase COVID‐19 patients and 280 control individuals. The authors reported severe dyslipidaemia, affecting lipoprotein particle size and distribution, alongside central metabolism dysregulation and accumulation of ketone bodies in COVID‐19 patients with respect to controls [42]. This signature has been replicated across numerous worldwide cohorts [42, 43, 44, 45, 46, 47, 48, 49, 50, 51, 52, 53, 54], with levels of certain metabolites (such as phenylalanine and glucose) and lipoprotein‐related parameters (such as cholesterol, free cholesterol, and phospholipids associated with LDL5) found to be progressively dysregulated from asymptomatic to severe and fatal state of the disease [54]; notably, the main features are variant‐independent [54]. The consistency of the results of studies conducted by different groups worldwide is particularly significant, as it demonstrates the value of combining metabolomics and lipoproteomics for disease profiling and, more generally, the high reproducibility of NMR‐based metabolomic results, not only in well designed and controlled round‐robin campaigns but in real life and under the hectic situation imposed by the pandemic on the hospitals worldwide.

During the critical phase of the first wave of the pandemic there was a race to repurpose existing drugs. In this context, the work by Meoni et al., which employed NMR fingerprinting to monitor the changes induced by tocilizumab treatment in a small cohort of patients, is notable [44]. By measuring post‐treatment levels of metabolites that were significantly dysregulated during infection, the researchers observed that eight metabolites partially or completely reverted toward “healthy” levels. Although the concentrations of certain lipoproteins were also significantly affected by the treatment, they did not return to “healthy” values [44]. This initial observation is consistent with the later finding by the same laboratory that, during the healing process (after the acute phase), the recovery of the lipoproteome is significantly slower than that of the metabolome [43].

Following the global vaccination campaigns against COVID‐19, efforts have been made to evaluate via metabolomics/lipoproteomics the safety of the different vaccines and the possible correlations with their  immunogenicity [55, 56, 57, 58, 59]. The third paper that we have selected concerns the use of NMR to study the vaccine response in 43 immunosuppressed inflammatory bowel disease patients [60]. In this work, Alexander et al. integrated untargeted ^1^H NMR‐based faecal metabolomics with 16S rRNA sequencing and bile acid profiling (UHPLC‐MS) to explore whether gut microbial composition and related metabolome could explain the variable serological response to SARS‐CoV‐2 vaccination observed in this population [60]. The authors identified a distinct faecal metabolomic profile between patients showing antibody responses above and below the geometric mean of a wide population cohort. Specifically, higher levels of trimethylamine, isobutyrate, and ω‐muricholic acid were associated with stronger vaccine‐induced antibody responses, whereas succinate, phenylalanine, taurine, and conjugated bile acids (taurolithocholate and taurodeoxycholate) characterized weaker responses. A supervised OPLS‐DA model confirmed the separation between the two groups (R^2^Y = 0.26, Q^2^ = 0.15; CV‐ANOVA *p* = 0.038) [60].

### 3.3 Neurodegeneration

Approximately one tenth of the studies analyzed in this review and classified under the broad category of human health specifically address research questions related to neurodegenerative and neurological disorders. Neurodegeneration is strongly associated with alterations in amino acid metabolism, redox chemistry, and metabolic alterations associated with metal homeostasis [61, 62, 63], all of which can be effectively investigated through NMR‐based metabolomic profiling. By mapping these molecular and metabolic perturbations, predictive biomarkers as well as mechanistic insights into specific pathways can be pointed out. To illustrate these trends, we selected three representative and highly cited studies [64, 65, 66] that converge on a shared goal: to unravel biochemical and mechanistic underpinnings of neurodegenerative and neurological diseases (i.e., Alzheimer's disease and Parkinson's disease), highlighting different but complementary aspects of metabolomics.

Ashraf et al. [64] dissected iron dyshomeostasis and its connection with metabolic dysfunction in Alzheimer's disease using an integrated approach that combines protein and elemental analysis with ^1^H NMR based metabolomics of brain tissue. They analyzed aqueous and organic phase samples obtained by methanol–chloroform extraction collected from Alzheimer's disease (AD) deceased patients and controls (*n* = 7 per group). From their analysis, ferroptotic‐like alterations in AD emerged: in particular, iron dyshomeostasis, zinc decrement, increased expression of light‐chain subunit of the cystine/glutamate transporter and lipid peroxidation were observed. Metabolite levels, particularly of metabolites involved in neuronal function, were examined, and a significantly increased excitatory glutamate to inhibitory GABA ratio was found, suggesting enhanced glutamate‐induced excitotoxicity in AD. Furthermore, the levels of glutamine, *N*‐acetyl‐aspartate and hypoxanthine were found to be significantly decreased in AD tissues. Taken together, these findings suggest that therapies targeting ferroptosis may be potentially beneficial in AD.

Tynkkynen et al. [65] in turn aimed to leverage on metabolomics of human‐derived samples for translational applications to identify novel biomarkers. They applied serum metabolomics to profile eight prospective cohorts comprising 22 623 participants who were free of dementia at baseline, with the aim of identifying novel early biomarkers of incident dementia and AD. The study found that reduced levels of branched‐chain amino acids were associated with an increased risk of developing dementia and AD. These findings further support the potential prognostic value of valine in predicting cognitive decline [67, 68, 69], warranting additional investigation for its use in early risk stratification. Interestingly, the authors acknowledged that replication in independent cohorts is inherently challenging due to differences in metabolomics platforms (i.e., NMR and mass spectrometry), cohort characteristics, and sample collection procedures, which may limit the direct generalizability of the findings. This issue is of key relevance to metabolomics research and is further elaborated in Section 2.1.

Finally, in recent years, the integration of metabolomics with gut microbiota profiling has emerged as an increasingly important frontier in metabolomic research, as discussed in Section 3.1. Within this context, Tan et al. [66] approached Parkinson's disease through a multi‐omics lens, linking gut microbiome alterations with host faecal metabolomic signatures. They evaluated 104 Parkinson's disease patients together with 96 controls free of neurological disorders. Their findings provide valuable insights into gut–brain pathophysiology, highlighting specific microbial metabolites as mediators of neurodegeneration and as potential pharmacological targets to develop microbiome‐informed therapeutic strategies.

Interestingly, the studies of Tynkkynen et al. [65] and Tan et al. [66] combine metabolomics approaches using both NMR and MS techniques. This combination allows for a more comprehensive coverage of the metabolome, capturing complementary information that would be missed if only a single platform were used. Such integrative strategies underscore the growing importance of multi‐platform metabolomics in advancing our understanding of complex biological pathophysiological states such as neurodegenerative disorders.

### 3.4 Cardiovascular Diseases

Over the past decade, cardiovascular research has undergone a deep methodological transformation. The availability of high‐throughput metabolomics and lipoproteomics platforms, together with statistical modeling, has allowed investigators to move from classical clinical risk factors to ensembles of biochemical alterations that link metabolic dysregulation to cardiovascular diseases. By quantifying circulating metabolites and lipoprotein subclasses in large populations, these approaches capture the altered metabolic state that often precedes the overt disease. The three studies discussed here by Holmes et al. [70], Vignoli et al. [71]and Conners et al. [72] exemplify this approach. Each uses quantitative ^1^H NMR‐metabolomics to predict cardiovascular risk, yet they differ in the population analyzed, in the clinical focus, and in translational potential. Taken together, they illustrate perfectly both the potential and the current limitations of NMR metabolomics in cardiovascular research.

Holmes et al. [70] analyzed plasma samples from the China Kadoorie Biobank to determine how lipoprotein subclasses and small‐molecule metabolites in plasma relate to myocardial infarction, ischemic stroke, and haemorrhagic stroke. The scale of the study, comprising roughly 900 myocardial infarction cases, 1150 ischemic stroke cases, 1140 intracerebral haemorrhage cases and about 1466 matched controls, provides enough statistical power to map differences across the different groups. NMR analysis was performed using the high‐throughput NMR metabolomics platform described by Soininen et al. [73] that allows for the measurement of 255 markers including lipoproteins subfractions and small metabolites. As expected, the results indicate that VLDL, IDL, and LDL particle concentrations and triglycerides are positively associated with myocardial infarction and ischemic stroke, while cholesterol in HDL particles is inversely related. However, these patterns were not observed for haemorrhagic stroke, suggesting that for this disease the atherosclerotic process could be less influential. Conversely, inflammatory and energy‐related metabolites such as GlycA, ketone bodies, and glucose are positively associated with all the three diseases. Strengths included a large sample size and state of the art NMR analysis; however, the single‐time case‐control design and lack of adjustment for confounders like lifestyle or treatments limited the robustness of the results.

Vignoli and colleagues [71] explored metabolomics in myocardial infarction from a different clinical perspective, asking whether an NMR‐derived serum fingerprint can predict mortality after an acute myocardial infarction. They evaluated 978 patients from the AMI‐Florence II registry [74], for which the 2‐year mortality status was retrospectively available. Blood was sampled 24 to 48 h after percutaneous coronary intervention, and three types of one‐dimensional ^1^H NMR spectra per sample (1D NOESY, CPMG, and diffusion‐edited) were acquired using a Bruker 600 MHz instrument. The spectra were fed, without identifying metabolites, into a random forest machine learning classifier [75], following a typical untargeted fingerprinting approach [16]. A small training set was used to define the risk score, and then it was applied to a larger validation set, reporting a high predictive power (AUC of about 0.80) in discriminating survivors from deceased patients. Interestingly, the NMR fingerprint score (in particular that derived from 1D NOESY) demonstrated independent and orthogonal prognostic power with respect to clinical predictors, including the Global Registry of Acute Coronary Events (GRACE) risk score [76]. Consistently, a linear combination of the random forest NMR score and GRACE score increases the discrimination. Overall, this study demonstrates that an untargeted, NMR‐spectra‐based classifier can add prognostic information after acute myocardial infarction. However, some limitations should also be mentioned. First, sample collections were done exclusively in the acute phase of the disease, impairing the acquisition of data correlated with the biochemical mechanisms of the transition to the quiescent phase. Second, even though the data were replicated in a validation set, a totally independent cohort for validation is lacking.

Conners et al. [72] focused on another critical cardiovascular condition: heart failure, a complex, sometimes subtle, and possibly life threatening, clinical syndrome. Numerous studies aimed at defining the underlying metabolic derangements and at discovering possible biomarkers for a very early diagnosis of cardiac failure, including NMR metabolomic studies [77, 78]. The authors of this paper applied a composite NMR‐derived score, dubbed the Metabolic Vulnerability Index, to predict mortality in a cohort of 1,382 heart failure patients. EDTA plasma collected from patients at the time of enrolment were analyzed on the high‐throughput 400 MHz Vantera clinical analyzer platform using the NMR LipoProfile approach developed by Labcorp (Morrisville, NC). The index predicted all‐cause mortality in the 10‐years follow‐up, independently of ejection fraction and conventional scores. The index, built on NMR measures of GlycA, small‐HDL particles, branched‐chain amino acids, and citrate, exemplifies another attempt to translate NMR data into a clinically meaningful measure, but again further validations are needed.

All the three studies rely on quantitative NMR of blood—whose reproducibility, standardization, and easy setup make it suitable for large datasets—and employ robust statistical frameworks to extract clinically relevant information from high‐dimensional data. However, the details are different: the study by Holmes is larger in scale, and has a more epidemiologic view on the links among individual lipoprotein subclasses and vascular events; Vignoli et al. attempt to directly use NMR fingerprints for a prognostic score in the acute phase of myocardial infarction; Conners et al. try to compress a limited panel of markers into a single prognostic index for heart failure patients. Together, they emphasize that metabolomic and even more lipoprotein signatures reflect the systemic cardiometabolic status of the patients, and that the field has now matured and moved beyond the proof‐of‐concept scale, entering a stage where its integration into patient management practice is becoming a realistic and hopefully imminent scenario.

## 4 Food and Nutrition

### 4.1 Food Origin and Traceability

Within the broad field of food and nutrition, a significant number of studies have focused on the use of NMR‐based metabolomics for food authentication, geographical origin, and traceability. This analytical approach has proven particularly powerful in identifying the metabolic fingerprints that reflect the combined effects of raw material, processing, and origin [79, 80, 81, 82, 83, 84].

NMR‐based metabolomics, when integrated with data‐driven analytical strategies, can provide a molecular‐level traceability framework applicable across diverse food systems. Whether used to track variety and fermentation technology or to authenticate origin and post‐harvest practices, this integrative approach allows for the construction of robust chemical fingerprints that encode the interplay between environment, genetics, and technology.

**FIGURE 9:**
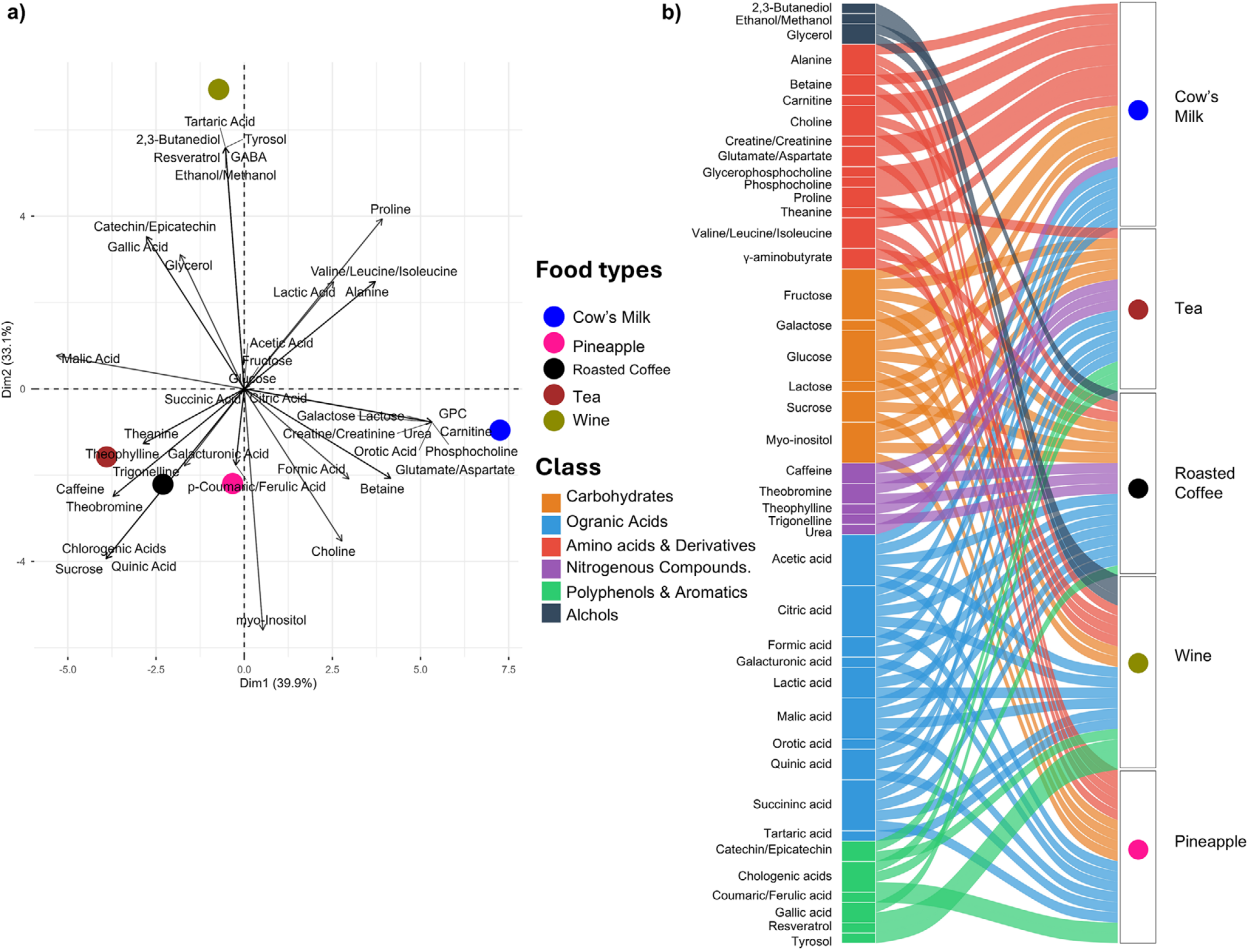
PCA and alluvial view of NMR‐detectable metabolites across food matrices. (a) PCA biplot summarizing similarities among the five matrices (cow's milk, tea, roasted coffee, wine, pineapple). Samples cluster according to overall metabolite composition in the score plot (colored dots); loadings (arrows) indicate metabolites driving separation along the first components. Each arrow represents a loading component, its length is proportional to the loading importance. (b) Alluvial representation linking metabolites (ordered and colored by compound class: carbohydrates, organic acids, amino acids & derivatives, nitrogenous compounds, polyphenols & aromatics, alcohols) to food matrices. Together, the plots highlight class‐level contributions to each matrix (e.g., polyphenols in tea/coffee; organic acids in wine) and shared NMR‐metabolites that bridge multiple foods.

In this context, the number and nature of metabolites quantified through NMR vary according to the specific food matrix and extraction method employed, yet all studies confirm the remarkable versatility of this technique. ^1^H NMR enables the simultaneous detection and quantification of a broad spectrum of low‐molecular‐weight compounds, from a dozen in roasted coffee extracts to nearly 40 in wines and more than 40 in black tea. These include amino acids, organic acids, sugars, polyphenols, alkaloids, and other molecules that collectively define the chemical identity of each product (Figure 9).

Importantly, in most of the cases analyses are conducted on extracts rather than raw matrices: hydroalcoholic, espresso, or moka preparations for coffee; buffered liquid samples for wine; and methanolic extracts for tea, ensuring optimal solubilization of polar metabolites and compatibility with deuterated solvents used in NMR spectroscopy.

The application of untargeted ^1^H NMR profiling to beverages of plant origin, such as wine, coffee, and tea (Figure 9), has provided remarkable insights into how compositional variability can be translated into predictive models of origin and quality (see next paragraph). The following examples illustrate the versatility of this approach across different plant‐based matrices.

Mascellani et al. [79] developed robust chemometric models to classify Czech wines according to type, grape variety, and geographical origin using an extensive dataset of 917 samples. The authors achieved a classification accuracy above 93% through machine learning, demonstrating the ability of NMR metabolomics to discriminate wines based on subtle compositional nuances related to variety and production area. A panel of discriminative metabolites was identified for each category: for wine type (color and sugar content), compounds like fructose, uridine, and catechins were significant; for grape variety, proline, phenylalanine, and epicatechins were key; and for geographical origin, succinic acid, glucose, and glycerol played a primary role. Regarding wine, NMR is also used to characterize the molecular signature of different winemaking practices, providing valuable insights into how production methods influence the final chemical composition and quality of the wine [82, 85].

A similar analytical rationale has been applied to coffee, another complex and economically relevant matrix. Ciaramelli et al. [80] employed NMR profiling to explore how coffee species, origin, and extraction procedures affect the bioactive composition of green and roasted coffee. Through a rapid NMR protocol, they quantified key metabolites, including chlorogenic acids, trigonelline, caffeine, and choline, linking their relative abundance to both geographical source and processing method. The study revealed that hydroalcoholic extraction maximized the recovery of low‐molecular‐weight compounds, particularly chlorogenic acids, whereas moka extraction enriched melanoidin content. Importantly, Arabica coffees exhibited a distinct metabolic profile characterized by a higher ratio of bioactive compounds to caffeine compared with Robusta [83].

Cui et al. [81] applied ^1^H NMR metabolomics integrated with machine learning algorithms to the classification of black teas according to geographical origin, variety, and processing type. By analysing 219 samples from multiple producing regions, the authors identified caffeine, malic acid, lysine, and β‐glucose as discriminants, achieving accuracies above 92% in origin prediction. Notably, tea variety emerged as a stronger determinant of metabolic fingerprint than processing, underscoring the biological basis of chemical differentiation.

### 4.2 Metabolomics in Human Nutrition and Food Quality

Recent research has increasingly expanded its use toward nutritional characterization and quality assessment of foods. NMR spectroscopy provides a molecular‐level view of nutritional composition, enabling the quantification and comparison of nutrients, bioactives, and metabolites that underpin both the quality and the functionality of food products. NMR‐based metabolomics now serves as a central analytical platform in molecular nutrition and food chemistry, bridging compositional analysis with the understanding of biochemical mechanisms that sustain nutritional value and health benefits [86, 87, 88, 89, 90, 91].

A paradigmatic example of NMR's application in nutritional food chemistry is the compositional characterization of commercial cow's milk [86]. In this study, 59 water‐soluble metabolites were identified in bovine milk using ^1^H NMR spectroscopy, encompassing sugars, amino acids, organic acids, nitrogenous compounds and alcohols (Figure 9). The work highlights how NMR‐based metabolomics can provide a precise and reproducible nutritional fingerprint of milk. Additionally, NMR metabolomics provides a molecular perspective on how nutritional composition varies among dairy products and processing types, as well as seasonal variation [92]. The same analytical rationale has been increasingly extended to human milk and plant‐based milk alternatives, revealing both compositional analogies and marked biochemical distances between animal and plant matrices [89, 90, 91]. Human and bovine milks are characterized by the presence of lactose, higher levels of citrate, and a wide range of amino acids such as glutamate and alanine, together with characteristic organic acids and short‐chain metabolites related to energy metabolism. In contrast, plant‐based milks, such as soy, oat, and almond, are typically enriched in sugars such as sucrose, glucose, and maltose and organic acids like malate and succinate. These compositional fingerprints reflect both the botanical origin and the technological processing involved, providing clear molecular markers for matrix differentiation.

In a broader nutritional context, Lang et al. [88] employed high‐field NMR, in combination with MS, to identify and structurally characterize atractyligenin‐derived glucuronide conjugates in human urine following coffee intake. This study exemplifies how NMR enables unambiguous structural elucidation of dietary metabolites, providing a molecular basis for the identification of biomarkers of food intake, for tracing metabolic pathways relevant to nutritional physiology, and to monitor interindividual variability in dietary metabolism, further linking analytical chemistry with nutritional science.

In a complementary direction, NMR metabolomics has also proven effective in characterizing bioactive metabolites within food by‐products, linking compositional profiling to functional assessment. In this context, the study by Azizan et al. [87] describes how to valorize pineapple residues, specifically the crown, peel, and core, by identifying phenolic acids, flavonoids, and organic acids responsible for the observed antioxidant and α‐glucosidase inhibitory activities. In this way, the same by‐products are not only recovered but also recognized as rich sources of functional molecules, demonstrating how metabolomics supports sustainable strategies for waste reduction and resource recovery in the food industry. This integrative use of NMR aligns with the principles of green and circular food chemistry, where compositional elucidation directly enables the transformation of waste streams into nutritionally and functionally valuable resources.

Collectively, these examples highlight how NMR‐based metabolomics bridges chemical compositional analysis and functional nutrition, offering a molecular‐level perspective on food quality.

## 5 Veterinary

### 5.1 Animal Health

Similarly to the study of human beings, metabolomics can analyze animal samples to provide insight into their metabolic state. This approach can be employed to study health, disease, genetics, and animal welfare [93]. However, there are a few peculiar challenges. When moving to “small” animals, the sample volume that can be collected can decrease significantly, which is problematic for NMR that usually requires a minimum volume of 160 µL to fill a 3 mm NMR tube. For farm animals or laboratory animals, genetics is complemented by homogeneity in nutrition and lifestyle, which on the one hand can facilitate the identification of characteristic fingerprints of pathophysiological states but on the other hand can lead to a loss of generality.

**FIGURE 10:**
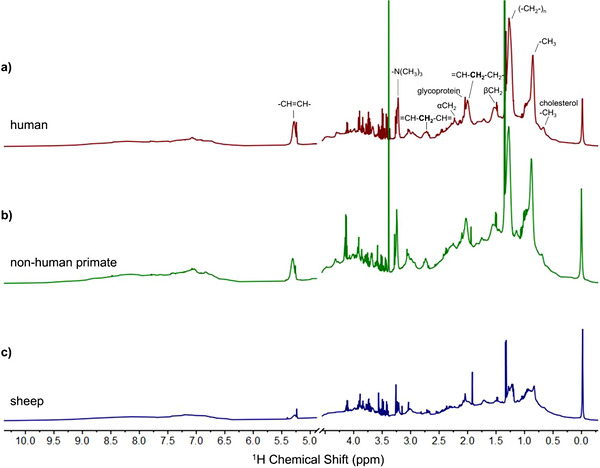
^1^H NMR spectra of serum samples collected from different mammals. The contributions of lipoprotein signals are annotated in the human samples.

Furthermore, we have seen in Section 3 that lipoproteins play an important role in the profiling of human pathologies. To date, there is no corresponding validated approach for the analysis of lipoproteins in different animals (Figure 10). For example, the Bruker In Vitro Diagnostics research (IVDr) Lipoprotein Subclass Analysis (B.I.‐LISA) platform provides an alternative to ultracentrifugation for human plasma and serum lipoprotein quantitation by applying statistical models calibrated on large human reference datasets. However, these models rely on the characteristic spectral features and lipoprotein composition of human samples. Since lipoprotein composition is inherently species‐specific, the direct application of these models to non‐human samples may lead to inaccurate quantitative interpretation. Therefore, although NMR spectra from animal samples clearly show species‐dependent lipoprotein patterns (Figure 10), dedicated calibration and validation against established reference methods would be required to enable reliable quantitative lipoprotein profiling in veterinary species. As a result, assessments in animal samples are limited to small molecule metabolites.

The most represented topics in our literature browsing involve bovine metabolomics, especially related to milk composition, energy balance, mastitis, and reproductive performance [94, 95, 96, 97]. In veterinary medicine, bovine respiratory disease (BRD) is a complex and multifactorial disease that represents one of the main health issues of welfare and economic significance. Indeed approximately 60%–90% of the morbidity and mortality that occurs in feedlots has been attributed to BRD [98, 99, 100]. However, the diagnosis of BRD remains challenging, as the methods currently applied are often subjective and lack accuracy. Blakebrough‐Hall et al. [101] applied untargeted ^1^H NMR‐based metabolomics of blood plasma to identify animals with BRD. The models identified 12 signals of importance in differentiating BRD and non‐BRD animals; eight out of twelve belong to phenylalanine, lactate, glutamine, hydroxybutyrate, tyrosine, citrate and leucine (the other four were unassigned).

Xu et al. [102] employed a combined ^1^H NMR and LC‐MS metabolomic approach to investigate negative energy balance (NEB) in dairy cows. The transition from pregnancy to lactation represents a critical physiological phase characterized by profound metabolic adaptations, during which the high energy demands of milk production coincide with a relatively low dry matter intake. Through milk metabolomic profiling of 27 LC‐MS measured metabolites and 35 NMR measured metabolites, the authors provided mechanistic biochemical insights into the metabolic consequences of NEB, including enhanced synthesis of nucleic acids and cell membrane phospholipids, altered protein glycosylation, and perturbations in one‐carbon and lipid metabolism.

Another topic well represented in our selection is the use of NMR‐based metabolomics for marine species welfare and precision aquaculture. Huynh et al. [103] investigated the effects of a combination of probiotics and prebiotics on the immune response of white shrimp (*Litopenaeus vannamei*) by analyzing hepatopancreas metabolites. The elevation of several key metabolites, including inosine monophosphate, valine, and betaine, indicates that symbiotic supplementation significantly enhances shrimp immunity, thereby improving the health and disease resistance of *L. vannamei*.

The three selected works collectively illustrate how metabolomics can translate complex pathophysiological phenomena into measurable chemical signatures, providing an integrative framework that links diet, environment, metabolism, and health outcomes.

### 5.2 Animal Nutrition

Animal nutrition and health management plays a central role in veterinary science, ensuring that feeding strategies support both animal welfare and the production of safe, high‐quality foods of animal origin. Metabolomics provides deep insights into how nutrition influences the quality and composition of animal‐derived products [92]. The following studies [104, 105, 106] illustrate how NMR‐based metabolomics can link dietary manipulation and farm practices to metabolic responses and product characteristics, highlighting its potential as a research and monitoring tool in animal production systems.

In the dairy sector, the two selected studies [104, 105] clearly demonstrate that the metabolic profile of milk is significantly affected by the feeding regimen of cows. O'Callaghan et al. [104] reported that animal nutrition markedly alters the rumen fluid metabolome, although it does not substantially influence the rumen microbiota composition. In the study by Tenori et al. [105] metabolomics proved crucial for molecular characterization of milk linking seasonal variations and the impact of specific feed types on the final product.

Similarly, in poultry science, metabolomics has been employed to optimize feed formulations. Broiler chickens have been selected for decades to increase feed efficiency and enhance breast meat yield. This selection process has led to significant changes in their nutritional requirements, particularly with regard to amino acids and protein, for example, to support their extraordinary breast muscle development [107]. Zampiglia et al. [106] by analyzing the plasma and muscle metabolome of broiler chickens demonstrated a clear separation between animal groups according to the dietary supplementation of arginine. Indeed, increasing the level of dietary arginine is significantly associated with the increase of plasma concentration of arginine and leucine and breast muscle arginine and acetate. Conversely, the levels of plasma proline, glutamate, acetoacetate, and adenosine and the levels of breast muscle acetone and inosine were reduced. Taken together, increased digestible arginine to lysine ratio (probably by modulating energy and protein metabolism) has a positive impact on feed efficiency without showing any negative effect on chicken mortality, breast muscle myopathies and meat quality attributes.

## 6 Plants

Two main research directions emerge among metabolomics studies focusing on plant systems: those addressing the chemical and physiological responses of plants to abiotic stress and those exploring plant–microbe interactions. In both contexts, metabolomics provides an understanding at a molecular level of how plants adapt to changing environmental conditions. Most of the studies use complementary analytical approaches (NMR and MS) to describe the metabolic reprogramming that occurs in plants subjected to diverse forms of stress. The combined use of the two analytical platforms stems from the challenges posed by the chemical complexity of plant matrices.

**FIGURE 11:**
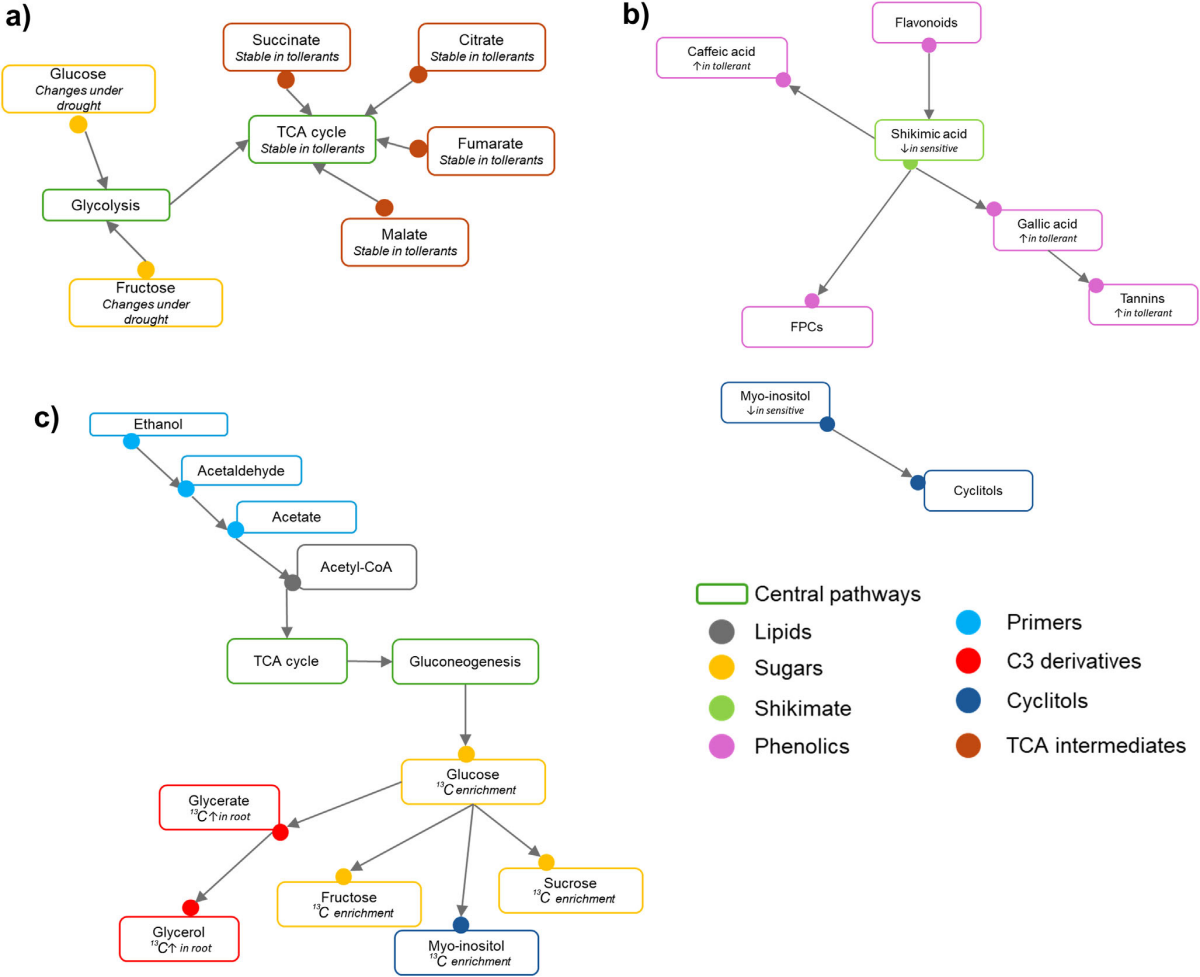
Overview of plant metabolic responses to drought stress. (a) *Thymus spp*. changes in soluble sugars (glucose, fructose) and TCA‐cycle intermediates (citrate, malate, fumarate, succinate) discriminate tolerant from sensitive genotypes. (b) *Eucalyptus spp*. tolerant clones accumulate antioxidant phenolics (gallic and caffeic acids, tannins, flavonoids, FPCs), while sensitive ones show decreased glucose, myo‐inositol and shikimic acid, indicating metabolic flux from the shikimate to FPCs pathway. (c) Ethanol‐primed plants: exogenous ethanol is oxidized to acetate and converted to acetyl‐CoA, feeding the TCA cycle and gluconeogenesis, resulting in ^1^ ^3^C enrichment of sugars (glucose, fructose and sucrose) and osmoprotectants (glycerate, glycerol, and myo‐inositol).

In *Thymus vulgaris* and *Thymus kotschyanus*, Ashrafi et al. [108] applied ^1^H NMR metabolomics to characterize drought‐induced metabolic adjustments in genotypes differing in tolerance (Figure 11a). Drought stress markedly affected sugars (higher levels of glucose and fructose), amino acids (higher levels of choline and alanine), and tricarboxylic acid (TCA) intermediates (higher levels of citric, malic, fumaric, and succinic acids), which emerged as key discriminants between tolerant and sensitive plants. The tolerant *T. kotschyanus* maintained higher relative water content, elevated chlorophyll levels, and a more stable TCA‐cycle profile, indicating that efficient carbon flux through central metabolism supports homeostasis and photosynthetic activity during water deficit. These findings emphasize the role of central carbon metabolism and osmoprotective amino acids in sustaining energy balance and drought resilience.

In *Eucalyptus spp*., Noleto‐Dias et al. [109] integrated UPLC‐MS and ^1^H NMR to investigate the chemical responses of 13 clones grown under well‐watered and water‐deficit conditions. Over 100 metabolites were annotated, including cyclitols, phenolic acids, flavonoids, formylated phloroglucinol compounds (FPCs) and fatty acids. The multi‐platform dataset captured both primary (NMR) and secondary metabolism (MS) metabolic processes, enabling the discrimination of genotypes according to their tolerance to drought (Figure 11b). Tolerant clones accumulated arginine, gallic and caffeic acids, and tannins, whereas sensitive clones showed reduced glucose, inositol and shikimic acids. This genotype‐dependent metabolic plasticity defines a biochemical signature of tolerance characterized by osmoprotective amino acids and antioxidant phenolic compounds that counteract oxidative stress and preserve cellular integrity. Such metabolites represent potential biomarkers for selecting drought‐resistant genotypes and targets for the metabolic engineering of stress resilience.

Bashir et al. [110] explored the biochemical mechanisms underlying ethanol‐induced drought tolerance in *Arabidopsis thaliana*, rice, and wheat, demonstrating that exogenous ethanol functions as a chemical priming agent, enhancing both signalling and metabolic resilience. Ethanol treatment activated abscisic acid (ABA)‐dependent pathways, leading to stomatal closure and induction of stress‐responsive genes via the PYR/PYL/RCAR‐PP2C‐SnRK2 (Pyrabactin Resistance/Pyrabactin Resistance‐Like/Regulatory Component of ABA Receptors‐Type 2C Protein Phosphatase‐Sucrose Non‐fermenting 1‐Related Protein Kinase 2) cascade. Using ^13^C‐labeled ethanol and NMR spectroscopy, the authors traced the incorporation of ethanol‐derived carbon into key metabolites of *A. thaliana*. Root and leaf analyses revealed ^13^C enrichment in glucose, fructose, and sucrose, while roots also contained labeled glycerate, glycerol, and myo‐inositol (Figure 11c). These results confirmed the assimilation of ethanol into central carbon metabolism. Biochemically, ethanol is first oxidized to acetaldehyde and acetic acid, then converted to acetyl‐CoA, which feeds into the TCA cycle and gluconeogenesis. This process promotes sugar accumulation and osmoprotection under drought conditions. The study demonstrates that ethanol acts as both a metabolic substrate and as a signalling modulator, reinforcing ABA‐mediated stress responses while sustaining carbon flux and energy homeostasis. This dual role establishes a pre‐adaptive metabolic state that improves drought tolerance.

Together, these studies illustrate that plant drought tolerance relies on the coordinated regulation of carbon–nitrogen metabolism, antioxidant phenolics, and central energy pathways.

## 7 Environment

### 7.1 Micro‐ and Nano‐Plastics

Within the broader field of environmental toxicology, traditional approaches have largely focused on measuring contaminant burdens and correlating them with physiological endpoints such as mortality or growth inhibition. However, at the environmentally relevant, low‐dose exposures typical of modern ecosystems, such overt responses are often absent or masked by biological variability. In this context, NMR‐based metabolomics has emerged as a transformative analytical tool capable of detecting subtle, sub‐lethal biochemical perturbations that precede visible toxicity in tissues or biofluids [111]. This systems‐level approach provides molecular evidence of stress, energetic imbalance, or oxidative load long before phenotypic effects become apparent, thereby bridging the gap between exposure and adverse outcome. Recent ecotoxicology studies employing NMR metabolomics have extended this rationale to one of the most pervasive classes of emerging contaminants, micro‐ and nano‐plastics (MP and NP).

Cappello et al. [112] applied ^1^H NMR profiling to the digestive glands of mussels (*Mytilus galloprovincialis*) exposed to 3 µm polystyrene MP. The analyses revealed time‐dependent metabolic variations between exposed and controls. These variations include: alterations in amino acid metabolism (leucine, isoleucine, valine, alanine, and tyrosine), indicative of systemic stress signalling; osmolyte imbalance (taurine, betaine, and hypotaurine), reflecting impaired cellular osmoregulation; antioxidant defence (glutathione depletion), consistent with oxidative stress; and central energy metabolism (glucose, glycogen, lactate, succinate, and malonate), suggesting mitochondrial dysfunction. These results demonstrate that even short‐term (24 h) MP exposure induces pronounced biochemical stress in mussels, a sentinel species for coastal ecosystems, and raise concerns about potential effects on food quality and trophic transfer.

In soil ecosystems, ^1^H NMR‐based metabolomics uncovered an equally marked biochemical interplay between MP and agrochemicals [113]. In *Eisenia fetida* (a standard soil bioindicator earthworm), co‐exposure to MP and the pesticide dufulin increased pesticide bioaccumulation—determined with the Bio‐Soil Accumulation Factor, peaking at day 14—and amplified oxidative stress, as shown by elevated malondialdehyde (a lipid peroxidation product), elevated superoxide dismutase activity, and depletion of glutathione (GSH). An expanded metabolic perturbation, including elevated leucine, isoleucine, valine, alanine, arginine, methionine, cysteine and inositol, alongside with depletion of glucose, glycine, choline, phenylalanine, tyrosine, and tryptophan, was observed. Pathway enrichment highlighted phenylalanine–tyrosine–tryptophan biosynthesis and phenylalanine metabolism as primary dufulin targets, with glycine–serine–threonine metabolism uniquely activated in the presence of MP. This metabolic profiling demonstrates that MP act as chemical vectors and metabolic amplifiers, enhancing pesticide‐induced toxicity in soil organisms.

In a mammalian developmental model, Mercer et al. [114] used ^1^H high resolution magic angle spinning (HR‐MAS) NMR spectroscopy to examine the foetal brain of mice after maternal ingestion (healthy pregnant mice) of 50 nm particles of polystyrene NP. The metabolomic profiles showed significant reductions in GABA (−40%), creatine (−21%) and glucose (−30%), together with sex‐dependent changes in asparagine. These data reveal that exposure to NP disrupts the balance of neurotransmitters, energy buffering, and central carbon metabolism in the developing brain, emphasizing the systemic reach of NP‐induced biochemical stress.

Collectively, these NMR‐based studies converge on a shared chemical signature of plastic‐induced toxicity: disruption of amino acid homeostasis, redox balance (GSH), and energy metabolism (glycolysis/TCA intermediates). By providing quantitative, non‐destructive, and pathway‐resolved insight into organismal metabolism, NMR metabolomics emerges as a key analytical platform for elucidating the molecular mechanisms and environmental impacts of MP and NP pollution, from aquatic bioindicators to soil invertebrates and vertebrate developmental models.

### 7.2 Per‐ and Polyfluoroalkyl Substances

Within the broader framework of persistent organic pollutants, per‐ and polyfluoroalkyl substances (PFAS) have attracted growing scientific and regulatory attention due to their extraordinary chemical stability, global ubiquity, and dangerous biological effects. The strong C─F bonds confer exceptional resistance to thermal, photochemical, and biological degradation, leading to their accumulation in water, soil, and biota, including humans. Conventional monitoring methods have mostly focused on targeted quantification of known PFAS congeners; however, recent advances in NMR spectroscopy have expanded the analytical and mechanistic understanding of these chemicals by enabling both molecular fingerprinting of fluorinated species and metabolomic profiling of biological responses [115]. Recent work illustrates how NMR closes the loop between source chemistry, environmental release, and biological effect for PFAS.

**FIGURE 12:**
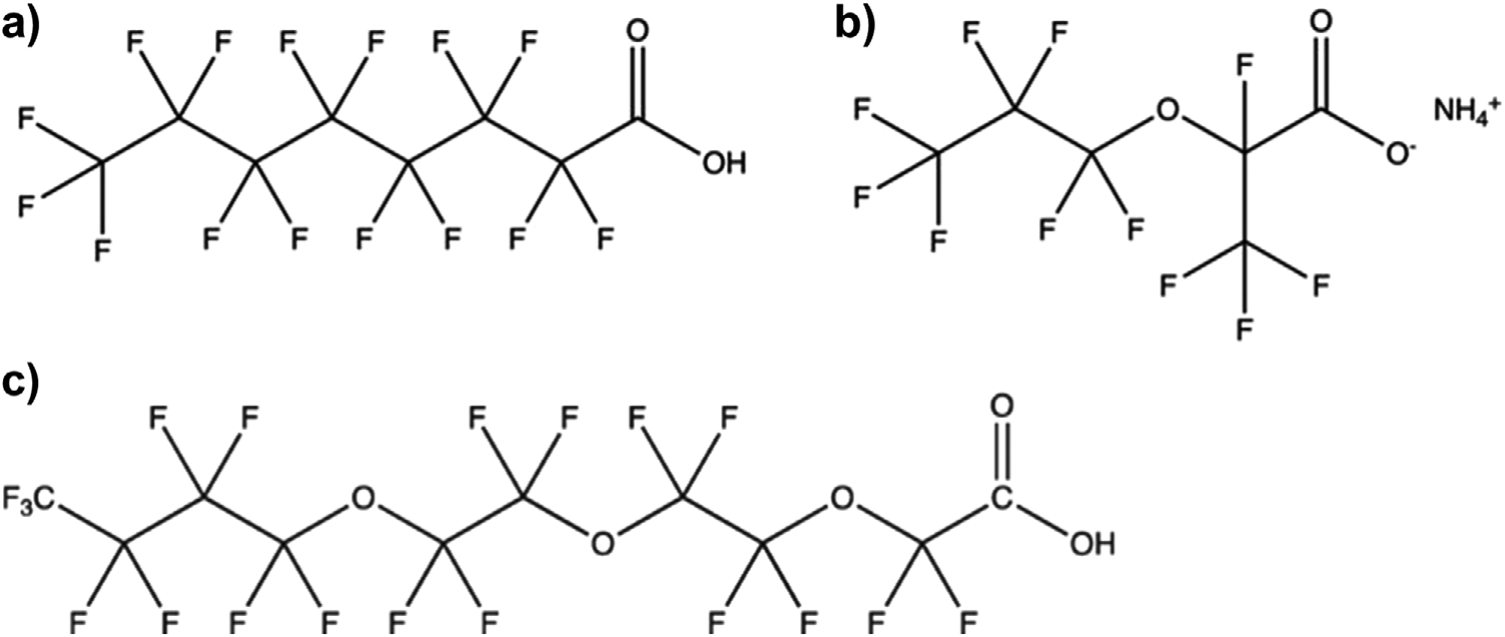
Chemical structures of the three PFAS evaluated in Gebreab et al. [116] (a) PFOA (perfluorooctanoic acid), a legacy long‐chain PFAS; b) GenX (ammonium 2,3,3,3‐tetrafluoro‐2‐(heptafluoropropoxy)propanoate), a next‐generation perfluoroether carboxylate; c) PFO3TDA (perfluoro(3,5‐dioxa‐7‐methyloctanoic) acid), another short‐chain perfluoroether carboxylic acid introduced as a replacement for legacy PFAS.

Gebreab et al. [116] investigated the metabolic and developmental toxicity of both legacy and next‐generation PFAS using zebrafish (*Danio rerio*) embryos as a vertebrate model system. The study compared perfluorooctanoic acid (PFOA), a well‐known legacy PFAS, with two newer perfluoroether carboxylic acids (PFECAs), GenX, and PFO3TDA (Figure 12), which have been introduced as supposedly safer alternatives. Embryos were exposed to increasing concentrations of each PFAS to evaluate acute toxicity (lethality), developmental malformations, and locomotor behavior.

Following exposure, intact embryo tissues were analyzed using ^1^H HR‐MAS spectroscopy. HR‐MAS analysis revealed profound, concentration‐dependent perturbations: decreased ATP, phosphocreatine, succinate, and lactate indicated mitochondrial dysfunction and impaired oxidative phosphorylation (energy metabolism); increased oxidized glutathione and depleted reduced glutathione reflected a redox imbalance and activation of the antioxidant defence system (oxidative stress); alterations in phosphocholine, glycerophosphocholine, and other choline derivatives pointed to membrane remodeling and potential activation of peroxisome proliferator‐activated receptors (PPAR)‐mediated lipid metabolism; perturbed glutamate, GABA, and taurine levels suggested neurotoxicity and disruption of early neuronal development; dysregulation of alanine, valine, and glutamine indicated cellular stress and hepatic metabolic alterations (amino acid metabolism). The integrated NMR‐derived metabolic fingerprints demonstrated that both GenX and PFO3TDA elicited biochemical disruptions comparable to PFOA, affecting the same metabolic axes with similar severity. These findings challenge the assumption that next‐generation PFAS are intrinsically safer and instead suggest a shared mechanism of toxicity involving mitochondrial dysfunction, oxidative stress, and PPAR‐mediated lipid remodeling.

At the population level, Faquih et al. [117] integrated LC‐MS/MS quantification of PFAS in plasma with ^1^H NMR lipidomics across two large European cohorts, Netherlands (584 subjects) and Germany (1,962 subjects). Using the Nightingale platform, 228 lipid‐related parameters were profiled, including total lipids, cholesterol fractions, phospholipids, triglycerides, and subclass‐resolved lipoproteins (HDL, LDL, IDL, VLDL). Multivariate regression analyses revealed a dose‐dependent positive association between PFAS levels (PFOA, PFOS, PFHxS) and circulating lipids across all lipoprotein subclasses, with stronger effects in participants ≤ 54 years. Remarkably, PFHxS, often marketed as a “safer” short‐chain replacement, exhibited similar lipid‐elevating properties to PFOS and PFOA. These findings point to a systemic remodeling of lipid homeostasis, likely involving PPAR activation and oxidative stress, and highlight the ability of NMR lipidomics to detect subtle metabolic consequences of chronic PFAS exposure also in humans.

Joudan et al. [118] used ^19^F NMR spectroscopy and targeted LC‐MS/MS to investigate the aqueous leaching of ultrashort‐chain PFAS from various polymeric materials. Remarkably, both fluorinated (PTFE, PFA, FEP) and non‐fluorinated plastics (PVC, PP, PEEK) release measurable amounts of trifluoroacetic acid, perfluoropropanoic acid and perfluorobutanoic acid into water. The occurrence of PFAS in non‐fluorinated matrices indicates contamination from manufacturing additives, catalyst residues, or degradation by‐products, while batch variability underscores the influence of industrial synthesis and processing chemistry on leaching behavior. Analytically, the study highlights the strengths of ^19^F NMR as a direct, quantitative, and non‐destructive tool for fluorine‐specific analysis. The distinct resonance patterns of fluorinated compounds provide molecular fingerprints that enable identification of both known and novel PFAS species. Combined with LC‐MS/MS confirmation, this approach delivers a comprehensive picture of total organofluorine content in polymer–water systems. These findings challenge the presumed inertness of fluoropolymers and reveal previously unrecognized sources of PFAS contamination relevant to both laboratory and environmental settings.

Although centered on materials rather than organisms, the study conceptually aligns with NMR‐based metabolomics, extending the principle of molecular fingerprinting from biological response to pollutant source characterization, thereby linking chemical origin, environmental persistence and biological impact within a single spectroscopic framework.

Collectively, these complementary studies illustrate how NMR spectroscopy unites molecular chemistry with systems toxicology. While ^19^F NMR offers direct structural and quantitative information on fluorinated species in complex matrices, ^1^H NMR‐based metabolomics and lipidomics elucidate the downstream biochemical consequences of exposure. By bridging chemical persistence with metabolic disruption, NMR provides a chemically grounded, systems‐level understanding of PFAS fate and toxicity, affirming its role in modern environmental chemistry.

## 8 Conclusion and Outlook

This review has outlined the key advances and achievements in NMR‐based metabolomics from 2018 to April 2025, identifying six main metabolomic topics (human health, food and nutrition, veterinary, plants, environment, and analytical methods). Statistical analyses deserve a special comment: at variance with other research areas, AI and machine learning methods were integrated into metabolomics workflows well before 2018 (as described in our previous review [15]) and are nowadays routinely employed. They will probably define the next development trajectory of metabolomics, moving beyond routine application toward AI‐driven data interpretation.

In the present review, we applied a macro‐scale statistical survey coupled with a micro‐scale critical evaluation of 42 key methodological and clinical breakthroughs. This contribution is intended as a rigorous analysis of the current state‐of‐the‐art. Our goal is not to provide long‐term predictions, but to offer points of reflection and identify emerging patterns that are currently shaping NMR‐based metabolomics.

A trend that is becoming increasingly clear is the translational potential of metabolomics in human health. This transition into clinical practice is driven by several factors: the availability of multicenter studies with large cohorts and, where possible, longitudinal data (baseline and follow‐up), which enable the creation of robust, disease‐specific metabolomic profiles. NMR spectroscopy offers the unique advantage of simultaneously quantifying a wide range of metabolites and numerous lipoprotein subfractions within a single sample, thereby helping to elucidate the critical interplay between the human metabolome and the gut microbiota. Furthermore, this progress is supported by the growing availability of comprehensive databases for metabolite identification, along with specialized tools and services for their precise quantification, and by the availability of low‐cost, easy‐to‐use/maintain benchtop instruments.

Of all these characteristics, only some can be transferred to other fields of research. For example, analogous studies on animals (laboratory animals as disease models; pets and farm animals for veterinary applications) are hindered by the lack of databases of analogous degree of completeness and by the lack of a relationship between lipoprotein particles and corresponding spectral components.

Benchtop NMR represents a valuable option in food analysis for rapid, non‐destructive quality control, to verify authenticity and monitor product development and nutritional quality. NMR's potential to surpass MS in the field of foodomics is increased by the “portability” of these instruments.

In plant studies, the use of NMR metabolomics has the power to determine the chemical structure of compounds and to differentiate isomers of compounds (both at the level of primary and secondary metabolites). Working with plants also brings the possibility of labeling substrates with NMR‐active heteronuclei (typically ^13^C) to investigate metabolite pathways.

The fields of plants, animals, and nutrition obviously overlap. The classification of the studies we have chosen refers to what appears to be the main focus of each work, but it is impossible to completely separate the various aspects.

Our examples also show an overlap between topics related to plants and animals and those concerning the environment. Here, we have mainly discussed plants in relation to plant resistance to drought (Section 6) or the effects of micro‐ and nanoplastic pollution on some simple marine or terrestrial organisms (Section 7).

Notably, ^19^F NMR is emerging as a powerful tool for environmental analysis, as it enables the detection and identification of fluorinated compounds and their degradation pathways. This application of ^19^F NMR can be broadly defined as a metabolomics approach due to two main features: it can be used as an untargeted approach without any prior knowledge of molecular structures, and it is related to small molecular components in complex matrices.

Last but not least, NMR spectroscopy is recognized as a green analytical technique from a methodological point of view [119]. Unlike many conventional methods, which often require large volumes of organic solvents, extensive sample preparation and multiple replicates, NMR spectroscopy minimizes the use of reagents and consumables. Furthermore, the benchtop version, which does not require the use of cryogenic liquids, has a significantly reduced environmental footprint. These characteristics make NMR an optimal platform to support the goals of green chemistry and sustainable research.

## 9 Methodological Approach

### 9.1 Data Collection

To start the process of data collection, we used Scopus to extract the DOI related to the articles of our interest, using the following query:

TITLE‐ABS‐KEY(metabolomic*) AND TITLE‐ABS‐KEY(“NMR” OR “nuclear magnetic resonance”) AND PUBYEAR > 2017 AND PUBYEAR < 2026 AND (LIMIT‐TO (DOCTYPE, “ar”) OR LIMIT‐TO (DOCTYPE, “le”) OR LIMIT‐TO (DOCTYPE,“sh”) OR LIMIT‐TO (DOCTYPE,“dp”)).

This query allowed us to select articles that contain, in the title or in the abstract, references to metabolomics and nuclear magnetic resonance (NMR) published between January 2018 and May 2025. Articles, letters, short communications and data papers were included, while other document types (for example reviews, conference proceedings or editorials) were excluded.

With these criteria, we created an initial dataset of 7008 DOIs.

The DOIs collected were grouped by publisher and, to optimize the next step of acquisition of texts, we selected the 14 publishers with the highest number of articles, for a total of 6091 DOIs. A further refinement was performed by three operators, who inspected the results to remove duplicates, MS articles, reviews, and other non‐pertinent contributions that were wrongly retrieved by the automated SCOPUS search. Therefore, the final number of analyzed articles was 5081.

The next step was to request and obtain the official APIs made available by some publishers, which we integrated in Python scripts to automate the download of the articles. Where these programmatic interfaces were available, documented and technically integrable, we preferred them to guarantee efficiency and scalability. In other cases, we used alternative procedures: on one side web scraping scripts developed ad hoc, always respecting the access and data use policy of each platform, and on the other side, when automated solutions were not possible, the manual download of articles.

All codes and procedures for data collection were developed in Python.

### 9.2 Pre‐Processing and Text Analysis

The articles acquired were submitted to pre‐processing and subsequent elaboration with Python scripts. The main goal was to automatically extract relevant information, in particular from the Methods section. When this section was not available or appeared incomplete, the extraction was extended to the full text of the article.

The pre‐processing pipeline consisted of several steps: normalization and cleaning of the text, application of text parsing procedures, extensive use of regular expressions for targeted identification of the information of interest, and finally organization of the data into a structured format (CSV). These steps made it possible to build a uniform dataset, easy to query and ready for subsequent analyses.

A further challenge encountered during this phase was the heterogeneous formatting of the papers coming from different publishers. In fact, the structure of the articles is far from standardized: for example, keywords can be presented as bullet lists, embedded within the abstract, or placed in separate sections depending on the journal. Similarly, the Methods section does not always appear under the same heading but is labeled in a variety of ways (“Methods”, “Materials and Methods”, “Experimental Section”, “Methodology”, and others). This variability required the development of more flexible parsing rules and additional effort to ensure that the information could be consistently recognized across the entire dataset.

### 9.3 Article Classification and Reference Model

For the task of classification of the articles we used PubMedBERT [120], a language model based on BERT (Bidirectional Encoder Representations from Transformers) [121] architecture and pre‐trained specifically on millions of abstracts available in PubMed. This choice is motivated by the fact that training on biomedical scientific texts allows the model to better capture the specialized scientific terminology compared to generic models.

PubMedBERT was further fine‐tuned using a dataset composed of 825 articles. The articles were divided in six thematic categories: environment (150), food (150), human health (150), analytical methods (150), plants (120), veterinary (105).

The fine‐tuning was executed on a workstation with GPU NVIDIA GeForce RTX 4070, using the software environment: Python 3.8, PyTorch 2.4.1+cu121, Transformers 4.46.3, and Datasets 2.19.1.

The assignment of labels was made manually, based mainly on the title of the article; when the title was not enough for a correct classification, also the abstract was consulted. This approach allowed us to define the classes in a coherent way, giving a solid base for the fine‐tuning of the model.

The texts were submitted to a minimal preliminary cleaning, that included removal of residual HTML/XML markups, elimination of non‐informative introductory strings (for example “BACKGROUND:”), and normalization of spaces. Subsequently, title and abstract were concatenated in one single text field, used as input for the model.

No balancing methods were applied; the model was trained directly on the data as such.

The fine‐tuned model was then applied for automatic classification of the 091 articles, assigning each article to the appropriate category based on the text content.

### 9.4 Train/Validation/Test Split

The cleaned and manually labeled dataset of 825 articles was divided in three subsets, maintaining the distribution of classes. The split was 70% training, 15% validation and 15% test.

The PubMedBERT model was then fine‐tuned, the texts were tokenized with the relative tokenizer, with truncation at 512 tokens. Training was done with these main hyperparameters:

- batch size = 8
- epochs = 6
- learning rate = 2 × 10^−^^5^
- weight decay = 0.01

The evaluation was done at each epoch on the validation set, with selection of the best model based on macro F1 score. Early stopping was also used, if no improvements were observed for three consecutive epochs, to reduce the risk of overfitting.

### 9.5 Final Evaluation and Metrics

The evaluation of the classifier based on PubMedBERT was performed using an independent test set, with the objective to measure classification performance and the reliability of the predicted probabilities. Considering the relatively small size of the test set (125 articles total), these results must be interpreted with caution, more as indication of general trend than as definitive and generalizable estimate of model performance (Table 2).

**TABLE 2:** Classification report (Test set, *n* = 125) – PubMedBERT. The numbers 0–5 indicate the six classes (human health, environment, plants, veterinary, food, analytical methods.

Class	Precision	Recall	F1‐score	Total per class
0	0.917	0.957	0.936	23
1	0.85	0.773	0.81	22
2	0.905	0.826	0.864	23
3	0.913	0.955	0.933	22
4	0.842	0.889	0.865	18
5	0.882	0.938	0.909	16
Accuracy	0.887	0.887	0.887	—
macro avg	0.885	0.889	0.886	—
weighted avg	0.887	0.887	0.886	—

The table shows the metrics calculated on the test set. Overall accuracy is 0.887, with average values of precision, recall, and F1‐score very similar (macro avg ≈ 0.886), confirming a good stability across the different categories. Analytical methods (0) and human health (3) obtained the highest scores (F1> 0.93), followed by veterinary (5, F1 = 0.909), while food (2) and plants (4) show intermediate values (F1 ≈ 0.86). The environment class (1) is the most critical (F1 = 0.810, recall = 0.773), indicating more difficulty in recognition. These differences between classes are illustrated in the following graphical analyses.

**FIGURE 13:**
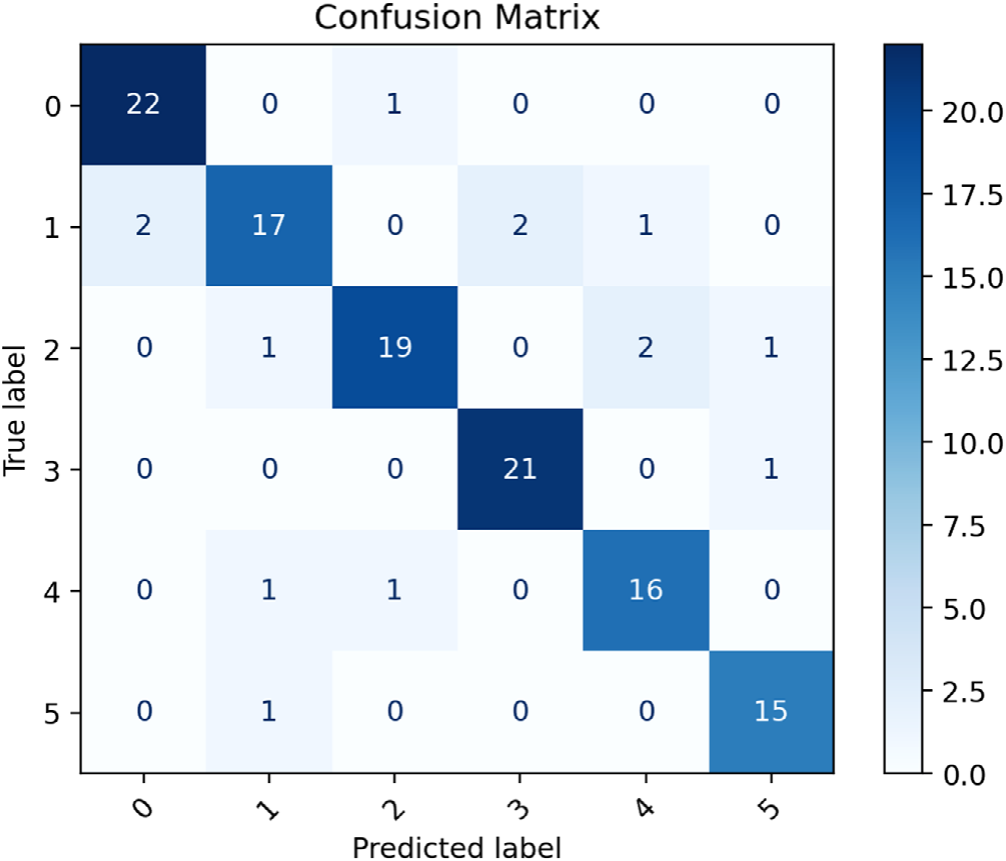
Confusion matrix of the PubMedBERT classifier.

The confusion matrix (Figure 13) gives a detailed overview of the distribution of predictions among the six considered categories. Most of the instances were classified correctly along the diagonal, showing a good general capacity of the model to distinguish the different domains. The classes analytical methods (0), human health (3) and veterinary (5) are among the most recognizable, with a very limited number of errors.

Some errors are instead observed between semantically close classes, in particular between environment (1) and food (2), and also between food (2) and plants (4). Overall, the matrix suggests a behavior coherent with the quantitative metrics, but it must be considered that the small size of the test set can amplify the impact of single errors on the final percentages.

**FIGURE 14:**
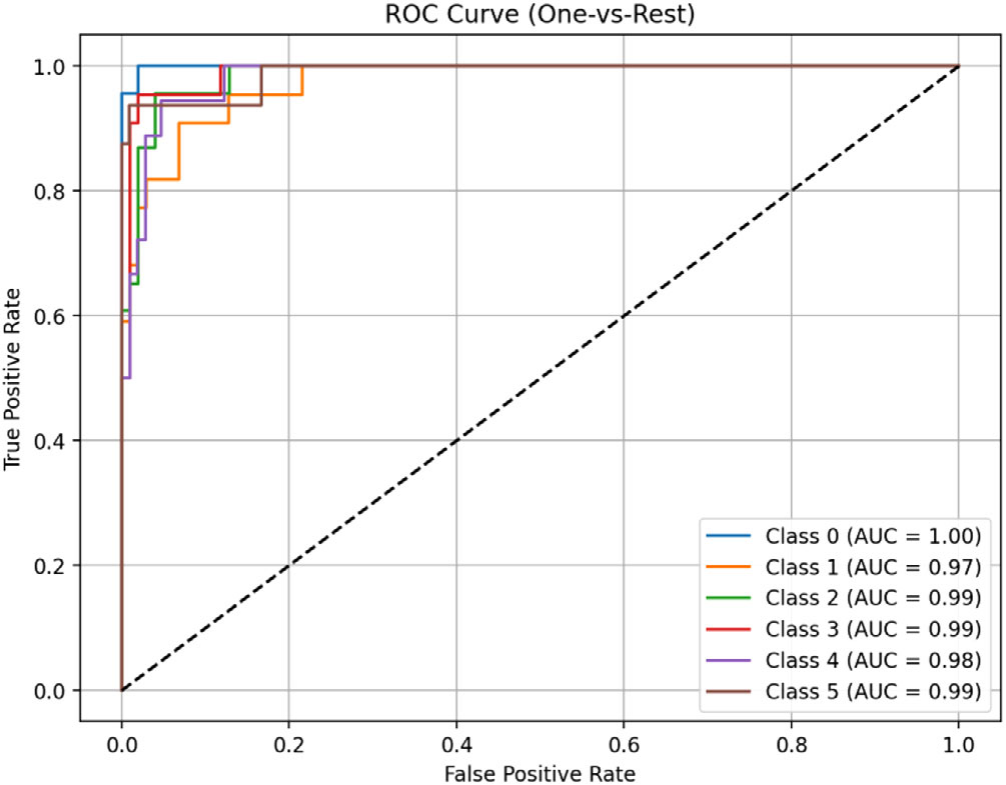
ROC curves and AUC values for each class.

The ROC curves (Figure 14) show high AUC values for all the classes, between 0.97 and 1.00. In particular, analytical methods (0), food (2), human health (3) and veterinary (5) reach values equal or higher than 0.99, while environment (1) and plants (4) are at 0.97 and 0.98, respectively. These results suggest a good discriminating capacity of the model also on unseen data, with slightly stronger performance for the classes that have more specific terminology.

**FIGURE 15:**
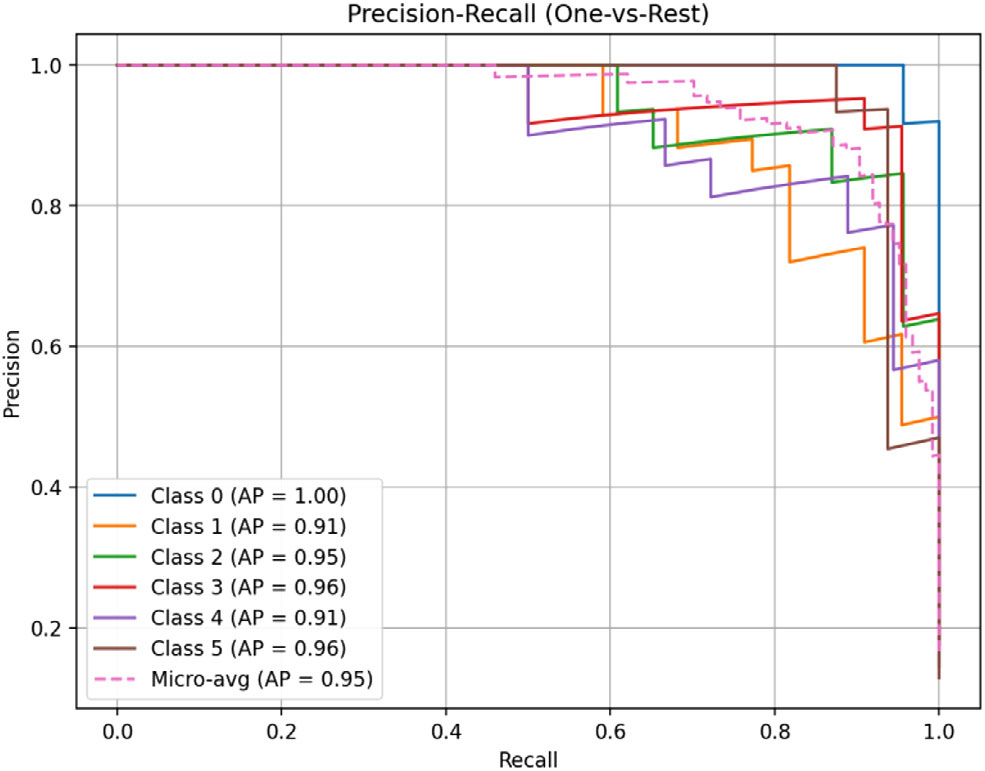
Precision‐recall curves and average precision (AP) values.

Considering the moderate imbalance of the dataset, Precision–Recall curves (Figure 15) [122] were also calculated, which allowed us to evaluate in a more accurate way the performance on minority classes through the calculation of average precision (AP).

The AP values are between 0.91 and 1.00: analytical methods (0) reached the maximum value (1.00), while food (2), human health (3) and veterinary (5) are at high values (≥ 0.95). The categories environment (1) and plants (4) show AP = 0.91, a bit lower but still indicative of a good recognition capacity. The micro‐avg curve (AP = 0.95) summarizes the overall trend of the model.

## Conflicts of Interest

The authors declare no conflicts of interest.

## Data Availability

Research data are not shared.

## References

[1] D. Broadhurst , R. Goodacre , S. N. Reinke , et al., “Guidelines and Considerations for the Use of System Suitability and Quality Control Samples in Mass Spectrometry Assays Applied in Untargeted Clinical Metabolomic Studies,” Metabolomics 14 (2018): 72, 10.1007/s11306-018-1367-3. 29805336 PMC5960010

[2] A.‐H. Emwas , R. Roy , R. T. McKay , et al., “NMR Spectroscopy for Metabolomics Research,” Metabolites 9 (2019): 123, 10.3390/metabo9070123. 31252628 PMC6680826

[3] P. G. Takis , V. Ghini , L. Tenori , P. Turano , and C. Luchinat , “Uniqueness of the NMR Approach to Metabolomics,” TrAC Trends in Analytical Chemistry 120 (2019): 115300, 10.1016/j.trac.2018.10.036.

[4] L. Tenori , P. Turano , and C. Luchinat , eMagRes (American Cancer Society, 2020), 199–204.

[5] D. J. Beale , F. R. Pinu , K. A. Kouremenos , et al., “Review of Recent Developments in GC–MS Approaches to Metabolomics‐based Research,” Metabolomics 14 (2018): 152, 10.1007/s11306-018-1449-2. 30830421

[6] L. Cui , H. Lu , and Y. H. Lee , “Challenges and Emergent Solutions for LC‐MS/MS Based Untargeted Metabolomics in Diseases,” Mass Spectrometry Reviews 37 (2018): 772–792, 10.1002/mas.21562. 29486047

[7] M. Jacob , A. L. Lopata , M. Dasouki , and A. M. Abdel Rahman , “Metabolomics Toward Personalized Medicine,” Mass Spectrometry Reviews 38 (2019): 221–238, 10.1002/mas.21548. 29073341

[8] S. Li , Y. Tian , P. Jiang , Y. Lin , X. Liu , and H. Yang , “Recent Advances in the Application of Metabolomics for Food Safety Control and Food Quality Analyses,” Critical Reviews in Food Science and Nutrition 61 (2021): 1448–1469, 10.1080/10408398.2020.1761287. 32441547

[9] G. Nannini , G. Meoni , A. Amedei , and L. Tenori , “Metabolomics Profile in Gastrointestinal Cancers: Update and Future Perspectives,” World Journal of Gastroenterology 26 (2020): 2514–2532, 10.3748/wjg.v26.i20.2514. 32523308 PMC7265149

[10] A. Vignoli and L. Tenori , “NMR‐based Metabolomics in Alzheimer's Disease Research: A Review,” Frontiers in Molecular Biosciences 10 (2023): 1308500, 10.3389/fmolb.2023.1308500. 38099198 PMC10720579

[11] A. Vignoli , G. Meoni , V. Ghini , et al., “NMR‐Based Metabolomics to Evaluate Individual Response to Treatments,” Handbook of Experimental Pharmacology 277 (Springer, 2022): 209–245, 10.1007/164_2022_618. 36318327

[12] A.‐H. M. Emwas , R. M. Salek , J. L. Griffin , and J. Merzaban , “NMR‐based Metabolomics in Human Disease Diagnosis: Applications, Limitations, and Recommendations,” Metabolomics 9 (2013): 1048–1072, 10.1007/s11306-013-0524-y.

[13] D. S. Wishart , “Emerging Applications of Metabolomics in Drug Discovery and Precision Medicine,” Nature Reviews Drug Discovery 15 (2016): 473–484, 10.1038/nrd.2016.32. 26965202

[14] G. Baima , M. Corana , G. Iaderosa , et al., “Metabolomics of Gingival Crevicular Fluid to Identify Biomarkers for Periodontitis: A Systematic Review With Meta‐analysis,” Journal of Periodontal Research 56 (2021): 633–645, 10.1111/jre.12872. 33710624

[15] A. Vignoli , V. Ghini , G. Meoni , et al., “High‐Throughput Metabolomics by 1D NMR,” Angewandte Chemie International Edition 58 (2019): 968–994, 10.1002/anie.201804736. 29999221 PMC6391965

[16] V. Ghini , G. Meoni , A. Vignoli , et al., “Fingerprinting and Profiling in Metabolomics of Biosamples,” Progress in Nuclear Magnetic Resonance Spectroscopy 138–139 (2023): 105–135, 10.1016/j.pnmrs.2023.10.002. 38065666

[17] B. Kamlage , S. G. Maldonado , B. Bethan , et al., “Quality Markers Addressing Preanalytical Variations of Blood and Plasma Processing Identified by Broad and Targeted Metabolite Profiling,” Clinical Chemistry 60 (2014): 399–412, 10.1373/clinchem.2013.211979. 24305685

[18] A.‐H. Emwas , R. Roy , R. T. McKay , et al., “Recommendations and Standardization of Biomarker Quantification Using NMR‐Based Metabolomics With Particular Focus on Urinary Analysis,” Journal of Proteome Research 15 (2016): 360–373, 10.1021/acs.jproteome.5b00885. 26745651 PMC4865177

[19] H. A. Haijes , E. A. J. Willemse , J. Gerrits , et al., “Assessing the Pre‐Analytical Stability of Small‐Molecule Metabolites in Cerebrospinal Fluid Using Direct‐Infusion Metabolomics,” Metabolites 9 (2019): 236, 10.3390/metabo9100236. 31635433 PMC6835587

[20] R. Lehmann , “From Bedside to Bench‐practical Considerations to Avoid Pre‐analytical Pitfalls and Assess Sample Quality for High‐resolution Metabolomics and Lipidomics Analyses of Body Fluids,” Analytical and Bioanalytical Chemistry 413 (2021): 5567–5585, 10.1007/s00216-021-03450-0. 34159398 PMC8410705

[21] T. Buergel , J. Steinfeldt , G. Ruyoga , et al., “Metabolomic Profiles Predict Individual Multidisease Outcomes,” Nature Medicine 28 (2022): 2309–2320, 10.1038/s41591-022-01980-3. PMC9671812 36138150

[22] J. Sotelo‐Orozco , S.‐Y. Chen , I. Hertz‐Picciotto , and C. M. Slupsky , “A Comparison of Serum and Plasma Blood Collection Tubes for the Integration of Epidemiological and Metabolomics Data,” Frontiers in Molecular Biosciences 8 (2021): 682134, 10.3389/fmolb.2021.682134. 34307452 PMC8295687

[23] A. Vignoli , L. Tenori , C. Morsiani , P. Turano , M. Capri , and C. Luchinat , “Serum or Plasma (and Which Plasma), That Is the Question,” Journal of Proteome Research 21 (2022): 1061–1072, 10.1021/acs.jproteome.1c00935. 35271285 PMC8981325

[24] A. Dey , B. Charrier , E. Martineau , et al., “Hyperpolarized NMR Metabolomics at Natural 13C Abundance,” Analytical Chemistry 92 (2020): 14867–14871, 10.1021/acs.analchem.0c03510. 33136383 PMC7705890

[25] D.‐W. Li , A. L. Hansen , C. Yuan , L. Bruschweiler‐Li , and R. Brüschweiler , “DEEP Picker is a Deep Neural Network for Accurate Deconvolution of Complex Two‐dimensional NMR Spectra,” Nature Communications 12 (2021): 5229, 10.1038/s41467-021-25496-5. PMC8410766 34471142

[26] P. G. Takis , H. Schäfer , M. Spraul , and C. Luchinat , “Deconvoluting Interrelationships Between Concentrations and Chemical Shifts in Urine Provides a Powerful Analysis Tool,” Nature Communications 8 (2017): 1662, 10.1038/s41467-017-01587-0. PMC5698486 29162796

[27] B. Khakimov , N. Mobaraki , A. Trimigno , V. Aru , and S. B. Engelsen , “Signature Mapping (SigMa): An Efficient Approach for Processing Complex Human Urine 1H NMR Metabolomics Data,” Analytica Chimica Acta 1108 (2020): 142–151, 10.1016/j.aca.2020.02.025. 32222235

[28] F. Savorani , G. Tomasi , and S. B. Engelsen , “Icoshift: A Versatile Tool for the Rapid Alignment of 1D NMR Spectra,” Journal of Magnetic Resonance 202 (2010): 190–202, 10.1016/j.jmr.2009.11.012. 20004603

[29] F. A. A. Mulder , L. Tenori , C. Licari , and C. Luchinat , “Practical Considerations for Rapid and Quantitative NMR‐based Metabolomics,” Journal of Magnetic Resonance 352 (2023): 107462, 10.1016/j.jmr.2023.107462. 37141802

[30] D. S. Wishart , Y. D. Feunang , A. Marcu , et al., “HMDB 4.0: The Human Metabolome Database for 2018,” Nucleic Acids Research 46 (2018): D608–D617, 10.1093/nar/gkx1089. 29140435 PMC5753273

[31] C. Knox , M. Wilson , C. M. Klinger , et al., “DrugBank 6.0: The DrugBank Knowledgebase for 2024,” Nucleic Acids Research 52 (2023): D1265–D1275, 10.1093/nar/gkad976. PMC10767804 37953279

[32] D. S. Wishart , E. Oler , H. Peters , et al., “MiMeDB: The Human Microbial Metabolome Database,” Nucleic Acids Research 51 (2022): D611–D620, 10.1093/nar/gkac868. PMC9825614 36215042

[33] Y. Fan and O. Pedersen , “Gut Microbiota in Human Metabolic Health and Disease,” Nature Reviews Microbiology 19 (2021): 55–71, 10.1038/s41579-020-0433-9. 32887946

[34] A. Vich Vila , S. Hu , S. Andreu‐Sánchez , et al., “Faecal Metabolome and its Determinants in Inflammatory Bowel Disease,” Gut 72 (2023): 1472–1485, 10.1136/gutjnl-2022-328048. 36958817 PMC10359577

[35] K. Deng , J.‐J. Xu , L. Shen , et al., “Comparison of Fecal and Blood Metabolome Reveals Inconsistent Associations of the Gut Microbiota With Cardiometabolic Diseases,” Nature Communications 14 (2023): 571, 10.1038/s41467-023-36256-y. PMC9894915 36732517

[36] N. Karu , L. Deng , M. Slae , et al., “A Review on Human Fecal Metabolomics: Methods, Applications and the Human Fecal Metabolome Database,” Analytica Chimica Acta 1030 (2018): 1–24, 10.1016/j.aca.2018.05.031. 30032758

[37] A. Botticelli , P. Vernocchi , F. Marini , et al., “Gut Metabolomics Profiling of Non‐small Cell Lung Cancer (NSCLC) Patients Under Immunotherapy Treatment,” Journal of Translational Medicine 18 (2020): 49, 10.1186/s12967-020-02231-0. 32014010 PMC6998840

[38] V. Ghini , L. Laera , B. Fantechi , et al., “Metabolomics to Assess Response to Immune Checkpoint Inhibitors in Patients With Non‐small‐cell Lung Cancer,” Cancers 12 (2020): 3574, 10.3390/cancers12123574. 33265926 PMC7760033

[39] Y. Talmor‐Barkan , N. Bar , A. A. Shaul , et al., “Metabolomic and Microbiome Profiling Reveals Personalized Risk Factors for Coronary Artery Disease,” Nature Medicine 28 (2022): 295–302, 10.1038/s41591-022-01686-6. PMC12365913 35177859

[40] J. Mayneris‐Perxachs , M. Cardellini , L. Hoyles , et al., “Iron Status Influences Non‐alcoholic Fatty Liver Disease in Obesity Through the Gut Microbiome,” Microbiome 9 (2021): 104, 10.1186/s40168-021-01052-7. 33962692 PMC8106161

[41] D. Vojinovic , D. Radjabzadeh , A. Kurilshikov , et al., “Relationship Between Gut Microbiota and Circulating Metabolites in Population‐based Cohorts,” Nature Communications 10 (2019): 5813, 10.1038/s41467-019-13721-1. PMC6925111 31862950

[42] C. Bruzzone , M. Bizkarguenaga , R. Gil‐Redondo , et al., “SARS‐CoV‐2 Infection Dysregulates the Metabolomic and Lipidomic Profiles of Serum,” Iscience 23 (2020): 101645, 10.1016/j.isci.2020.101645. 33043283 PMC7534591

[43] V. Ghini , G. Meoni , L. Pelagatti , et al., “Profiling Metabolites and Lipoproteins in COMETA, an Italian Cohort of COVID‐19 Patients,” PLOS Pathogens 18 (2022): e1010443, 10.1371/journal.ppat.1010443. 35446921 PMC9022834

[44] G. Meoni , V. Ghini , L. Maggi , et al., “Metabolomic/Lipidomic Profiling of COVID‐19 and Individual Response to Tocilizumab,” PLOS Pathogens 17 (2021): e1009243, 10.1371/journal.ppat.1009243. 33524041 PMC7877736

[45] T. Kimhofer , S. Lodge , L. Whiley , et al., “Integrative Modelling of Quantitative Plasma Lipoprotein, Metabolic and Amino Acid Data Reveals a Multi‐organ Pathological Signature of SARS‐CoV‐2 Infection,” Journal of Proteome Research 19 (2020): 4442–4454, 10.1021/acs.jproteome.0c00519. 32806897

[46] S. Lodge , P. Nitschke , T. Kimhofer , et al., “NMR Spectroscopic Windows on the Systemic Effects of SARS‐CoV‐2 Infection on Plasma Lipoproteins and Metabolites in Relation to Circulating Cytokines,” Journal of Proteome Research 20 (2021): 1382–1396, 10.1021/acs.jproteome.0c00876. 33426894

[47] R. A. Ballout , H. Kong , M. Sampson , et al., “The NIH Lipo‐COVID Study: A Pilot NMR Investigation of Lipoprotein Subfractions and Other Metabolites in Patients With Severe COVID‐19,” Biomedicines 9 (2021): 1090, 10.3390/biomedicines9091090. 34572275 PMC8471250

[48] R. Masuda , S. Lodge , P. Nitschke , et al., “Integrative Modeling of Plasma Metabolic and Lipoprotein Biomarkers of SARS‐CoV‐2 Infection in Spanish and Australian COVID‐19 Patient Cohorts,” Journal of Proteome Research 20 (2021): 4139–4152, 10.1021/acs.jproteome.1c00458. 34251833

[49] H. Julkunen , A. Cichońska , P. E. Slagboom , and P. Würtz , “Metabolic Biomarker Profiling for Identification of Susceptibility to Severe Pneumonia and COVID‐19 in the General Population,” Elife 10 (2021): e63033, 10.7554/eLife.63033. 33942721 PMC8172246

[50] E. Baranovicova , A. Bobcakova , R. Vysehradsky , et al., “The Ability to Normalise Energy Metabolism in Advanced COVID‐19 Disease Seems to be One of the Key Factors Determining the Disease Progression—A Metabolomic NMR Study on Blood Plasma,” Applied Sciences 11 (2021): 4231, 10.3390/app11094231.

[51] M. Bizkarguenaga , C. Bruzzone , R. Gil‐Redondo , et al., “Uneven Metabolic and Lipidomic Profiles in Recovered COVID‐19 Patients as Investigated by Plasma NMR Metabolomics,” Nmr in Biomedicine 35 (2021): e4637, 10.1002/nbm.4637. 34708437 PMC8646702

[52] B. S. B. Correia , V. G. Ferreira , P. M. F. D. Piagge , et al., “qNMR‐Based Metabolomics Discrimination of Covid‐19 Severity,” Journal of Proteome Research 21 (2022): 1640–1653, 10.1021/acs.jproteome.1c00977. 35674498

[53] R. Gil‐Redondo , R. Conde , M. Bizkarguenaga , et al., “An NMR‐Based Model to Investigate the Metabolic Phenoreversion of COVID‐19 Patients Throughout a Longitudinal Study,” Metabolites 12 (2022): 1206, 10.3390/metabo12121206. 36557244 PMC9788519

[54] V. Ghini , W. Vieri , T. Celli , et al., “COVID‐19: A Complex Disease With a Unique Metabolic Signature,” Plos Pathogens 19 (2023): e1011787, 10.1371/journal.ppat.1011787. 37943960 PMC10662774

[55] V. Ghini , L. Maggi , A. Mazzoni , et al., “Serum NMR Profiling Reveals Differential Alterations in the Lipoproteome Induced by Pfizer‐BioNTech Vaccine in COVID‐19 Recovered Subjects and Naïve Subjects,” Frontiers in Molecular Biosciences 9 (2022): 839809, 10.3389/fmolb.2022.839809. 35480886 PMC9037139

[56] Y. Wang , X. Wang , L. D. W. Luu , et al., “Proteomic and Metabolomic Signatures Associated With the Immune Response in Healthy Individuals Immunized With an Inactivated SARS‐CoV‐2 Vaccine,” Frontiers in Immunology 13 (2022): 848961, 10.3389/fimmu.2022.848961. 35686122 PMC9171821

[57] J. Lang , A. Bernal , J. Wist , et al., “Longitudinal Study on Immunologic, Lipoproteomic, and Inflammatory Responses Indicates the Safety of Sequential COVID‐19 Vaccination,” Journal of Molecular Medicine 103 (2025): 421–433, 10.1007/s00109-025-02527-y. 40074874 PMC12003606

[58] H. I. Abufares , R. A. Zenati , N. C. Soares , et al., “A Non‐targeted Metabolomics Comparative Study on Plasma of Pfizer and Sinopharm COVID‐19 Vaccinated Individuals, Assessed by (TIMS‐QTOF) Mass Spectrometry,” Heliyon 10 (2024): e35443, 10.1016/j.heliyon.2024.e35443. 39170395 PMC11336712

[59] Y. Peng , L. Zhang , C. K. P. Mok , et al., “Baseline Gut Microbiota and Metabolome Predict Durable Immunogenicity to SARS‐CoV‐2 Vaccines,” Signal Transduction and Targeted Therapy 8 (2023): 373, 10.1038/s41392-023-01629-8. 37743379 PMC10518331

[60] J. L. Alexander , B. H. Mullish , N. P. Danckert , et al., “The Gut Microbiota and Metabolome are Associated With Diminished COVID‐19 Vaccine‐induced Antibody Responses in Immunosuppressed Inflammatory Bowel Disease Patients,” EBioMedicine 88 (2023): 104430, 10.1016/j.ebiom.2022.104430. 36634565 PMC9831064

[61] L. Chen , Q. Shen , Y. Liu , et al., “Homeostasis and Metabolism of Iron and Other Metal Ions in Neurodegenerative Diseases,” Signal Transduction and Targeted Therapy 10 (2025): 31, 10.1038/s41392-024-02071-0. 39894843 PMC11788444

[62] J. I. Sbodio , S. H. Snyder , and B. D. Paul , “Redox Mechanisms in Neurodegeneration: From Disease Outcomes to Therapeutic Opportunities,” Antioxidants & Redox Signaling 30 (2019): 1450–1499, 10.1089/ars.2017.7321. 29634350 PMC6393771

[63] N. Sanghai and G. K. Tranmer , “Biochemical and Molecular Pathways in Neurodegenerative Diseases: An Integrated View,” Cells 12 (2023): 2318, 10.3390/cells12182318. 37759540 PMC10527779

[64] A. Ashraf , J. Jeandriens , H. G. Parkes , and P.‐W. So , “Iron Dyshomeostasis, Lipid Peroxidation and Perturbed Expression of Cystine/Glutamate Antiporter in Alzheimer's Disease: Evidence of Ferroptosis,” Redox Biology 32 (2020): 101494, 10.1016/j.redox.2020.101494. 32199332 PMC7083890

[65] J. Tynkkynen , V. Chouraki , S. J. van der Lee , et al., “Association of Branched‐chain Amino Acids and Other Circulating Metabolites With Risk of Incident Dementia and Alzheimer's Disease: A Prospective Study in Eight Cohorts,” Alzheimers and Dementia 14 (2018): 723–733, 10.1016/j.jalz.2018.01.003. PMC6082422 29519576

[66] A. H. Tan , C. W. Chong , S.‐Y. Lim , et al., “Gut Microbial Ecosystem in Parkinson Disease: New Clinicobiological Insights From Multi‐Omics,” Annals of Neurology 89 (2021): 546–559, 10.1002/ana.25982. 33274480

[67] J. B. Toledo , M. Arnold , G. Kastenmüller , et al., “Metabolic Network Failures in Alzheimer's Disease: A Biochemical Road Map,” Alzheimer's & Dementia 13 (2017): 965–984, 10.1016/j.jalz.2017.01.020. PMC5866045 28341160

[68] A. Vignoli , S. Paciotti , L. Tenori , et al., “Fingerprinting Alzheimer's Disease by 1H Nuclear Magnetic Resonance Spectroscopy of Cerebrospinal Fluid,” Journal of Proteome Research 19 (2020): 1696–1705, 10.1021/acs.jproteome.9b00850. 32118444

[69] H. Basun , L. G. Forssell , O. Almkvist , et al., “Amino Acid Concentrations in Cerebrospinal Fluid and Plasma in Alzheimer's Disease and Healthy Control Subjects,” Journal of Neural Transmission General Section 2 (1990): 295–304, 10.1007/BF02252924. 2078309

[70] M. V. Holmes , I. Y. Millwood , C. Kartsonaki , et al., “Lipids, Lipoproteins, and Metabolites and Risk of Myocardial Infarction and Stroke,” Journal of the American College of Cardiology 71 (2018): 620–632, 10.1016/j.jacc.2017.12.006. 29420958 PMC5811927

[71] A. Vignoli , L. Tenori , B. Giusti , et al., “NMR‐based Metabolomics Identifies Patients at High Risk of Death Within Two Years After Acute Myocardial Infarction in the AMI‐Florence II Cohort,” BMC Medicine 17 (2019): 3, 10.1186/s12916-018-1240-2. 30616610 PMC6323789

[72] K. M. Conners , J. J. Shearer , J. Joo , et al., “The Metabolic Vulnerability Index: A Novel Marker for Mortality Prediction in Heart Failure,” JACC Heart Fail 12 (2024): 290–300, 10.1016/j.jchf.2023.06.013. 37480881 PMC10949384

[73] P. Soininen , A. J. Kangas , P. Würtz , T. Suna , and M. Ala‐Korpela , “Quantitative Serum Nuclear Magnetic Resonance Metabolomics in Cardiovascular Epidemiology and Genetics,” Circulation: Cardiovascular Genetics 8 (2015): 192–206. 25691689 10.1161/CIRCGENETICS.114.000216

[74] F. Cesari , R. Marcucci , A. M. Gori , et al., “Reticulated Platelets Predict Cardiovascular Death in Acute Coronary Syndrome Patients. Insights From the AMI‐Florence 2 Study,” Thrombosis and Haemostasis 109 (2013): 846–853, 10.1160/TH12-09-0709. 23494003

[75] L. Breiman , “Random Forests”, Machine Learning 45 (2001): 5–32.

[76] E. W. Tang , C.‐K. Wong , and P. Herbison , “Global Registry of Acute Coronary Events (GRACE) Hospital Discharge Risk Score Accurately Predicts Long‐term Mortality post Acute Coronary Syndrome,” American Heart Journal 153 (2007): 29–35, 10.1016/j.ahj.2006.10.004. 17174633

[77] L. Tenori , X. Hu , P. Pantaleo , et al., “Metabolomic Fingerprint of Heart Failure in Humans: A Nuclear Magnetic Resonance Spectroscopy Analysis,” International Journal of Cardiology 168 (2013): e113–e115, 10.1016/j.ijcard.2013.08.042. 24007967

[78] A. Vignoli , A. Fornaro , L. Tenori , et al., “Metabolomics Fingerprint Predicts Risk of Death in Dilated Cardiomyopathy and Heart Failure,” Frontiers in Cardiovascular Medicine 9 (2022), 10.3389/fcvm.2022.851905. PMC9021397 35463749

[79] A. Mascellani , G. Hoca , M. Babisz , P. Krska , P. Kloucek , and J. Havlik , “1H NMR Chemometric Models for Classification of Czech Wine Type and Variety,” Food Chemistry 339 (2021): 127852, 10.1016/j.foodchem.2020.127852. 32889133

[80] C. Ciaramelli , A. Palmioli , and C. Airoldi , “Coffee Variety, Origin and Extraction Procedure: Implications for Coffee Beneficial Effects on Human Health,” Food Chemistry 278 (2019): 47–55, 10.1016/j.foodchem.2018.11.063. 30583399

[81] C. Cui , Y. Xu , G. Jin , et al., “Machine Learning Applications for Identify the Geographical Origin, Variety and Processing of Black Tea Using 1H NMR Chemical Fingerprinting,” Food Control 148 (2023): 109686, 10.1016/j.foodcont.2023.109686.

[82] T. Martellini , L. Sposato , S. Pucci , et al., “Influence of In‐amphorae Vinification on the Molecular Profile of Sangiovese and Cabernet Franc,” Flavour and Fragrance Journal 37 (2022): 219–233, 10.1002/ffj.3697.

[83] G. Meoni , C. Luchinat , E. Gotti , A. Cadena , and L. Tenori , “Phenotyping Green and Roasted Beans of Nicaraguan Coffea Arabica Varieties Processed With Different Post‐harvest Practices,” Applied Sciences (Switzerland) 11 (2021): 11779, 10.3390/app112411779.

[84] G. Meoni , L. Tenori , F. Di Cesare , et al., “NMR‐based Metabolomic Approach to Estimate Chemical and Sensorial Profiles of Olive Oil,” Computational and Structural Biotechnology Journal 27 (2025): 1359–1369, 10.1016/j.csbj.2025.03.045. 40235639 PMC11999361

[85] A. R. Cordeiro , I. de Lacerda Bezerra , A. P. Santana‐Filho , P. R. Benedetti , M. Ingberman , and G. L. Sassaki , “Wine Fermentation Process Evaluation Through NMR Analysis: Polysaccharides, Ethanol Quantification and Biological Activity,” Food Chemistry 451 (2024): 139531, 10.1016/j.foodchem.2024.139531. 38704992

[86] A. Foroutan , A. C. Guo , R. Vazquez‐Fresno , et al., “Chemical Composition of Commercial Cow's Milk,” Journal of Agricultural and Food Chemistry 67 (2019): 4897–4914, 10.1021/acs.jafc.9b00204. 30994344

[87] A. Azizan , A. X. Lee , N. A. Abdul Hamid , et al., “Potentially Bioactive Metabolites From Pineapple Waste Extracts and Their Antioxidant and α‐Glucosidase Inhibitory Activities by 1H NMR,” Foods 9 (2020): 173, 10.3390/foods9020173. 32053982 PMC7073707

[88] R. Lang , A. Beusch , and S. Dirndorfer , “Metabolites of Dietary Atractyligenin Glucoside in Coffee Drinkers' Urine,” Food Chemistry 405 (2023): 135026, 10.1016/j.foodchem.2022.135026. 36442242

[89] G. Meoni , L. Tenori , and C. Luchinat , “Nuclear Magnetic Resonance‐Based Metabolomic Comparison of Breast Milk and Organic and Traditional Formula Milk Brands for Infants and Toddlers,” OMICS: A Journal of Integrative Biology 24 (2020): 424–436, 10.1089/omi.2019.0125. 32522087

[90] G. Meoni , L. Tenori , G. Niero , M. De Marchi , and C. Luchinat , “NMR‐Based Metabolomic Profiling Highlights Functional Nutritional Gaps Between Human Milk, Infant Formulas, and Animal Milks,” Metabolites 15 (2025): 620, 10.3390/metabo15090620. 41003006 PMC12471977

[91] G. Meoni , I. Sousa , L. Tenori , et al., “A Metabolic Profiling Approach to Characterize and Discriminate Plant‐based Beverages and Milk,” Journal of Dairy Science 108 (2025): 5675–5695, 10.3168/jds.2025-26332. 40252764

[92] G. Niero , G. Meoni , L. Tenori , et al., “Grazing Affects Metabolic Pattern of Individual Cow Milk,” Journal of Dairy Science 105 (2022): 9702–9712, 10.3168/jds.2022-22072. 36307248

[93] H. Tran , M. McConville , and P. Loukopoulos , “Metabolomics in the Study of Spontaneous Animal Diseases,” Journal of Veterinary Diagnostic Investigation 32 (2020): 635–647, 10.1177/1040638720948505. 32807042 PMC7488963

[94] T. Bobbo , G. Meoni , G. Niero , et al., “Nuclear Magnetic Resonance Spectroscopy to Investigate the Association Between Milk Metabolites and Udder Quarter Health Status in Dairy Cows,” Journal of Dairy Science 105 (2021): 535–548, S0022‐0302(21)00964–4. 34656344 10.3168/jds.2021-20906

[95] A. Basoglu , “Effects of Boron Supplementation on Peripartum Dairy Cows' Health,” Biological Trace Element Research 179 (2017): 218–225, 10.1007/s12011-017-0971-9. 28229388

[96] A. Basoglu , I. Sen , G. Meoni , L. Tenori , and A. Naseri , “NMR‐Based Plasma Metabolomics at Set Intervals in Newborn Dairy Calves With Severe Sepsis,” Mediators of Inflammation 2018 (2018): 1–12, 10.1155/2018/8016510. PMC5883973 29743812

[97] A. Basoglu , N. Baspinar , L. Tenori , C. Licari , and E. Gulersoy , “Nuclear Magnetic Resonance (NMR)‐based Metabolome Profile Evaluation in Dairy Cows With and Without Displaced Abomasum,” The Veterinary Quarterly 40 (2020): 1–15, 10.1080/01652176.2019.1707907. 31858882 PMC6968509

[98] P. M. V. Cusack , N. McMeniman , and I. J. Lean , “The Medicine and Epidemiology of Bovine Respiratory Disease in Feedlots,” Australian Veterinary Journal 81 (2003): 480–487, 10.1111/j.1751-0813.2003.tb13367.x. 15086084

[99] D. Mosier , “Review of BRD Pathogenesis: The Old and the New,” Animal Health Research Reviews 15 (2014): 166–168, 10.1017/S1466252314000176. 25351390

[100] A. Basoglu , N. Baspinar , L. Tenori , A. Vignoli , and R. Yildiz , “Plasma Metabolomics in Calves With Acute Bronchopneumonia,” Metabolomics 12 (2016): 128, 10.1007/s11306-016-1074-x.

[101] C. Blakebrough‐Hall , A. Dona , M. J. D'occhio , J. McMeniman , and L. A. González , “Diagnosis of Bovine Respiratory Disease in Feedlot Cattle Using Blood 1H NMR Metabolomics,” Scientific Reports 10 (2020): 115, 10.1038/s41598-019-56809-w. 31924818 PMC6954258

[102] W. Xu , A. van Knegsel , E. Saccenti , R. van Hoeij , B. Kemp , and J. Vervoort , “Metabolomics of Milk Reflects a Negative Energy Balance in Cows,” Journal of Proteome Research 19 (2020): 2942–2949, 10.1021/acs.jproteome.9b00706. 32633519 PMC7426013

[103] T.‐G. Huynh , A.‐C. Cheng , C.‐C. Chi , K.‐H. Chiu , and C.‐H. Liu , “A Synbiotic Improves the Immunity of White Shrimp, *Litopenaeus Vannamei*: Metabolomic Analysis Reveal Compelling Evidence,” Fish & Shellfish Immunology 79 (2018): 284–293, 10.1016/j.fsi.2018.05.031. 29778843

[104] T. F. O'Callaghan , R. Vázquez‐Fresno , A. Serra‐Cayuela , et al., “Pasture Feeding Changes the Bovine Rumen and Milk Metabolome,” Metabolites 8 (2018): 27, 10.3390/metabo8020027. 29642378 PMC6027121

[105] L. Tenori , C. Santucci , G. Meoni , V. Morrocchi , G. Matteucci , and C. Luchinat , “NMR Metabolomic Fingerprinting Distinguishes Milk From Different Farms,” Food Research International 113 (2018): 131–139, 10.1016/j.foodres.2018.06.066. 30195505

[106] M. Zampiga , L. Laghi , M. Petracci , et al., “Effect of Dietary Arginine to Lysine Ratios on Productive Performance, Meat Quality, Plasma and Muscle Metabolomics Profile in Fast‐growing Broiler Chickens,” Journal of Animal Science and Biotechnology 9 (2018): 79, 10.1186/s40104-018-0294-5. 30455879 PMC6223088

[107] W. A. Dozier , M. T. Kidd , and A. Corzo , “Dietary Amino Acid Responses of Broiler Chickens,” Journal of Applied Poultry Research 17 (2008): 157–167, 10.3382/japr.2007-00071.

[108] M. Ashrafi , M.‐R. Azimi‐Moqadam , P. Moradi , E. MohseniFard , F. Shekari , and M. Kompany‐Zareh , “Effect of Drought Stress on Metabolite Adjustments in Drought Tolerant and Sensitive Thyme,” Plant Physiology and Biochemistry 132 (2018): 391–399, 10.1016/j.plaphy.2018.09.009. 30286404

[109] C. Noleto‐Dias , E. A. de , T. Picoli , et al., “Metabolomics Characterizes Early Metabolic Changes and Markers of Tolerant *Eucalyptus* ssp. Clones Against Drought Stress,” Phytochemistry 212 (2023): 113715, 10.1016/j.phytochem.2023.113715. 37156433

[110] K. Bashir , D. Todaka , S. Rasheed , et al., “Ethanol‐Mediated Novel Survival Strategy Against Drought Stress in Plants,” Plant & Cell Physiology 63 (2022): 1181–1192, 10.1093/pcp/pcac114. 36003026 PMC9474946

[111] M. Watanabe , K. A. Meyer , T. M. Jackson , T. B. Schock , W. E. Johnson , and D. W. Bearden , “Application of NMR‐based Metabolomics for Environmental Assessment in the Great Lakes Using Zebra Mussel (Dreissena polymorpha),” Metabolomics 11 (2015): 1302–1315, 10.1007/s11306-015-0789-4. 26366138 PMC4559106

[112] T. Cappello , G. De Marco , G. Oliveri Conti , et al., “Time‐dependent Metabolic Disorders Induced by Short‐term Exposure to Polystyrene Microplastics in the Mediterranean Mussel *Mytilus Galloprovincialis* ,” Ecotoxicology and Environmental Safety 209 (2021): 111780, 10.1016/j.ecoenv.2020.111780. 33352432

[113] W. Sun , Z. Meng , R. Li , et al., “Joint Effects of Microplastic and Dufulin on Bioaccumulation, Oxidative Stress and Metabolic Profile of the Earthworm (*Eisenia fetida*),” Chemosphere 263 (2021): 128171, 10.1016/j.chemosphere.2020.128171. 33297140

[114] G. V. Mercer , N. E. Harvey , K. L. Steeves , et al., “Maternal Exposure to Polystyrene Nanoplastics Alters Fetal Brain Metabolism in Mice,” Metabolomics 19 (2023): 96, 10.1007/s11306-023-02061-3. 37989919

[115] S. O. Dauda , R. Rai , E. G. Cushman , A. Abel , D. Shimon , and L. B. Casabianca , “1H and 19F NMR Toolbox for Examining Interactions Between Fluorinated Compounds and Polystyrene Nanoparticles,” Journal of Physical Chemistry B 129 (2025): 5642–5651, 10.1021/acs.jpcb.5c02259. 40406892

[116] K. Y. Gebreab , M. N. H. Eeza , T. Bai , et al., “Comparative Toxicometabolomics of Perfluorooctanoic Acid (PFOA) and Next‐generation Perfluoroalkyl Substances,” Environmental Pollution 265 (2020): 114928, 10.1016/j.envpol.2020.114928. 32540561

[117] T. O. Faquih , E. N. Landstra , A. van Hylckama Vlieg , et al., “Per‐ and Polyfluoroalkyl Substances Concentrations are Associated With an Unfavorable Cardio‐Metabolic Risk Profile: Findings From Two Population‐Based Cohort Studies,” Exposure and Health 16 (2024): 1251–1262, 10.1007/s12403-023-00622-4.

[118] S. Joudan , J. Gauthier , S. A. Mabury , and C. J. Young , “Aqueous Leaching of Ultrashort‐Chain PFAS From (Fluoro)Polymers: Targeted and Nontargeted Analysis,” Environmental Science & Technology Letters 11 (2024): 237–242, 10.1021/acs.estlett.3c00797.

[119] K. A. Mielko , N. Pudełko‐Malik , A. Tarczewska , and P. Młynarz , “NMR Spectroscopy as a ‘Green Analytical Method’ in Metabolomics and Proteomics Studies,” Sustainable Chemistry and Pharmacy 22 (2021): 100474, 10.1016/j.scp.2021.100474.

[120] Y. Gu , R. Tinn , H. Cheng , et al., “Domain‐Specific Language Model Pretraining for Biomedical Natural Language Processing,” ACM Transactions on Computing for Healthcare 3 (2021): 1–23.

[121] J. Devlin , M.‐W. Chang , and K. Lee , Toutanova in *Proceedings of the 2019 Conference of the North American Chapter of the Association for Computational Linguistics: Human Language Technologies, Volume 1 (Long and Short Papers)* , ed. J. Burstein , C. Doran , and T. Solorio , (Association For Computational Linguistics, Minneapolis, Minnesota, 2019), 4171–4186.

[122] T. Saito and M. Rehmsmeier , “The Precision‐Recall Plot is More Informative Than the ROC Plot When Evaluating Binary Classifiers on Imbalanced Datasets,” PLoS ONE 10 (2015): e0118432, 10.1371/journal.pone.0118432. 25738806 PMC4349800

